# Partitioning net carbon dioxide fluxes into photosynthesis and respiration using neural networks

**DOI:** 10.1111/gcb.15203

**Published:** 2020-07-02

**Authors:** Gianluca Tramontana, Mirco Migliavacca, Martin Jung, Markus Reichstein, Trevor F. Keenan, Gustau Camps‐Valls, Jerome Ogee, Jochem Verrelst, Dario Papale

**Affiliations:** ^1^ DIBAF Department for Innovation in Biological Agro‐food and Forestry Systems University of Tuscia Viterbo Italy; ^2^ Image Processing Laboratory (IPL) Parc Científic Universitat de València Universitat de València Paterna Spain; ^3^ Max Planck Institute for Biogeochemistry Jena Germany; ^4^ Department of Environmental Science, Policy and Management UC Berkeley Berkeley CA USA; ^5^ Earth and Environmental Sciences Area Lawrence Berkeley National Lab Berkeley CA USA; ^6^ INRAE UMR 1391 ISPA Villenaved’Ornon France; ^7^ Euro‐Mediterranean Center on Climate Change (CMCC) Viterbo Italy

**Keywords:** carbon dioxide fluxes partitioning, ecosystem respiration (RECO), eddy covariance, gross primary production (GPP), machine learning, net ecosystem exchange, neural network

## Abstract

The eddy covariance (EC) technique is used to measure the net ecosystem exchange (NEE) of CO_2_ between ecosystems and the atmosphere, offering a unique opportunity to study ecosystem responses to climate change. NEE is the difference between the total CO_2_ release due to all respiration processes (RECO), and the gross carbon uptake by photosynthesis (GPP). These two gross CO_2_ fluxes are derived from EC measurements by applying partitioning methods that rely on physiologically based functional relationships with a limited number of environmental drivers. However, the partitioning methods applied in the global FLUXNET network of EC observations do not account for the multiple co‐acting factors that modulate GPP and RECO flux dynamics. To overcome this limitation, we developed a hybrid data‐driven approach based on combined neural networks (NN_C‐part_). NN_C‐part_ incorporates process knowledge by introducing a photosynthetic response based on the light‐use efficiency (LUE) concept, and uses a comprehensive dataset of soil and micrometeorological variables as fluxes drivers. We applied the method to 36 sites from the FLUXNET2015 dataset and found a high consistency in the results with those derived from other standard partitioning methods for both GPP (*R* ^2^ > .94) and RECO (*R* ^2^ > .8). High consistency was also found for (a) the diurnal and seasonal patterns of fluxes and (b) the ecosystem functional responses. NN_C‐part_ performed more realistic than the traditional methods for predicting additional patterns of gross CO_2_ fluxes, such as: (a) the GPP response to VPD, (b) direct effects of air temperature on GPP dynamics, (c) hysteresis in the diel cycle of gross CO_2_ fluxes, (d) the sensitivity of LUE to the diffuse to direct radiation ratio, and (e) the post rain respiration pulse after a long dry period. In conclusion, NN_C‐part_ is a valid data‐driven approach to provide GPP and RECO estimates and complementary to the existing partitioning methods.

## Graphical abstract

In this paper, we present an innovative neural network (NN_C‐part_) for partitioning net ecosystem exchange of CO_2_, measured by eddy covariance over land ecosystems, into ecosystem respiration (RECO) and gross primary production (GPP). NN_C‐part_ has a hybrid nature for assimilating the light‐use efficiency (LUE) concept. The GPP and RECO produced by NN_C‐part_ resulted consistent with estimates provided in FLUXNET2015 and patterns of fluxes very realistic, being the method able to reproduce: the GPP and RECO response to meteorological forces; hysteresis in the diel cycle of fluxes; effect of diffuse to direct radiation ratio on LUE, respiration pulse.

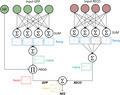

## 1 INTRODUCTION

The eddy covariance (EC) technique offers a unique opportunity for monitoring carbon and energy exchanges between land ecosystems and the atmosphere (Baldocchi, 2003) allowing near‐continuous measurements integrated at the ecosystem scale. The number of study sites equipped with EC systems has increased over the years (Baldocchi, 2014; Chu, Baldocchi, John, Wolf, & Reichstein, 2017; Pastorello et al., 2017), and now we estimate that more than 1,400 globally distributed sites (Chu et al., 2017) are monitoring the most representative land ecosystems in different climate conditions. Most of the EC study sites are organized in regional networks such ICOS, AmeriFlux, and AsiaFlux, and contribute to the global FLUXNET network (Baldocchi, 2014).

The EC method allows for the measurement of the net ecosystem exchange (NEE) which is the difference between two larger flux components: gross primary production (GPP) and ecosystem respiration (RECO). GPP is the gross amount of carbon uptake by photosynthesis from vegetation while RECO is the total carbon efflux by the respiration processes of all organisms. Estimating GPP and RECO is a key step to better understand the underlying mechanisms constraining ecosystem function. Moreover, GPP and RECO estimates from EC are useful for modeling, supporting process‐based model parameterization and validation, data assimilation, plant trait retrieval by model inversion (Dutta, Schimel, Sun, van der Tol, & Frankenberg, 2019; Pacheco‐Labrador et al., 2019), upscaling (Jung et al., 2020; Tramontana et al., 2016), as well as photosynthesis estimates based on remote sensing (e.g., Arnone et al., 2008; Verrelst et al., 2016; Zhang et al., 2016).

The EC technique does not directly measure GPP and RECO, and for this reason, numerical methods (termed partitioning methods) have been proposed for estimating GPP and RECO from NEE measurements (e.g., Desai et al., 2008; Keenan et al., 2019; Lasslop et al., 2010; Reichstein et al., 2005; Sulman, Tyler Roman, Scanlon, Wang, & Novick, 2016). The most widely used approaches are based on the use of NEE measurements for fitting simple physiologically based nonlinear relationships to estimate GPP and RECO using few meteorological drivers. These functional relationships are in general either light response functions linking global incoming radiation and GPP (Gilmanov et al., 2003), or respiration functions based on temperature (Reichstein et al., 2005), or also combinations of the two approaches (Keenan et al., 2019; Lasslop et al., 2010). The simple implementation and the robustness of the results (Lasslop et al., 2010) have led to their adoption as standard processing tools in the FLUXNET community (Pastorello et al., 2020, accepted).

However, these partitioning methods rely on important assumptions about the flux dynamics and their relationship with environmental drivers. Importantly, the assumed functional relations used can be similar to those applied in ecosystem models that make use of these data for their validation, creating a risk of circularity. In addition, although the functional relationships used in standard partitioning approaches are known to be valid at the organ level (where they can be measured), their direct application at the ecosystem spatial scale should be evaluated carefully (Medlyn, 1998; Medlyn et al., 2017; Musavi et al., 2016). Furthermore, to guarantee their wide applicability, partitioning methods use a limited number of drivers (i.e., air temperature, vapor pressure deficit, and global radiation). However, fluxes dynamics are also potentially affected by many important environmental factors, such as soil moisture, soil temperature, or the ratio of diffuse to direct radiation, among others, that are generally not considered in the partitioning methods (Lasslop et al., 2012; Wohlfahrt & Galvagno, 2017). These limitations are partially compensated by the use of short temporal moving windows for parameters estimation, which takes more slowly changing factors indirectly into consideration (such as phenology, water, or substrate availability), but does not handle fast ecosystem responses well, like for example respiration pulses following rain events (Williams, Hanan, Scholes, & Kutsch, 2009).

Some studies have attempted to solve the limitation of FLUXNET standard partitioning methods by developing more comprehensive approaches. For instance, Scanlon and Kustas (2010) coupled CO_2_ and H_2_O fluxes dynamics in the flux variance similarity approach for simultaneously partitioning carbon and water fluxes. The method, although interesting, requires canopy scale estimates of water‐use efficiency (WUE), which introduces assumptions and uncertainty. More recently, the estimation of gross CO_2_ fluxes from NEE has been approached using the EC method in combination with parallel measurements of a trace gas such as carbonyl sulfide (COS; Commane et al., 2015) or ^13^C isotopes (Ogée, Peylin, et al., 2003; Oikawa et al., 2017; Wehr et al., 2016; Wehr & Saleska, 2015) with the aim to disentangle the photosynthesis signals from respiration in daytime NEE measurements. Both methods are promising and are starting to be applied in the field; however, they currently require extensive and expensive instrumentation and the uncertainty in the results is still large (Oikawa et al., 2017; Whelan et al., 2018), limiting, for now, their application to a restricted number of study sites. These methods, however, are all subject to assumptions that could affect the resulting estimates of GPP and RECO. A possible alternative approach, based on existing measurements and not subject to the limitation of the standard methods, could be provided by machine learning methods.

Machine learning methods are generally used to model underlying complex relationships linking a set of predictors with one or more outputs. The core characteristics of machine learning methods are that predictors are not prescribed and the relationships between input (drivers) and output (fluxes) are inferred from the data. Several studies have reported the capability of machine learning to reproduce complex patterns in ecological studies, and in particular in relation to EC measurements (e.g., see Moffat, Beckstein, Churkina, Mund, & Heimann, 2010; Moffat et al., 2007; Papale & Valentini, 2003; Reichstein et al., 2019; Tramontana et al., 2016).

In this study, we develop and test an empirical machine learning‐based approach for retrieving GPP and RECO from NEE. The main motivation was to evaluate if a purely empirical approach could provide estimates of the two components without any predefined relationships and with the flexibility to use multiple sets of meteorological and biological forcings.

In this experiment, we used artificial neural networks (ANNs), which have already been tested as a partitioning tool in a cross‐site intercomparison among several partitioning methods (Desai et al., 2008; Oikawa et al., 2017), without appreciable differences with respect to the other methods. In those studies, the ANNs were trained using only nighttime measurements and then applied to extrapolate RECO during daytime. Here, we revisited the ANN implementation by proposing a new scheme in which the ANN is constrained using the measured net CO_2_ fluxes to directly estimate the two components GPP and RECO.

Our aim was to design a machine learning‐based method flexible enough to: (a) use a large set of drivers, accounting for the complexity of the GPP and RECO responses, (b) ensure general applicability of the method to allow ANN‐based partitioning in the context of FLUXNET thanks to the use of generally measured variables. In order to evaluate the results, we compared the GPP and RECO produced by the ANN with the estimates obtained by two partitioning methods used in FLUXNET, by analyzing: (a) the consistency of the estimates at yearly, seasonal, daily, and (half) hourly time steps; (b) the dynamics of the seasonal and diurnal cycles of the estimated gross CO_2_ fluxes; (c) the functional relationships between micrometeorological inputs and fluxes in output as reproduced by the ANN; and (d) the effectiveness of the proposed method to predict additional patterns of gross CO_2_ fluxes not (or less) accounted for by the standard partitioning methods.

## 2 MATERIALS AND METHODS

### 2.1 ANN algorithm

ANNs are nonlinear and nonparametric methods for regression and classification that artificially emulate the functioning of a biological brain (Haykin, 1999). The base unit of an ANN is the “neuron” where the numeric information in the input is weighted, condensed, and transformed (by a linear/nonlinear transfer/activation function) to be transferred to other neurons. Neurons are organized in layers that are interconnected: the outputs of *m* neurons in one layer are the inputs for *n* neurons of the next layer and the signals are transferred through connections associated with multiplicative weights. The learning (or training) process of an ANN consists of adjusting the weights of the network on the basis of specific examples provided as input (supervised training).

**FIGURE 1:**
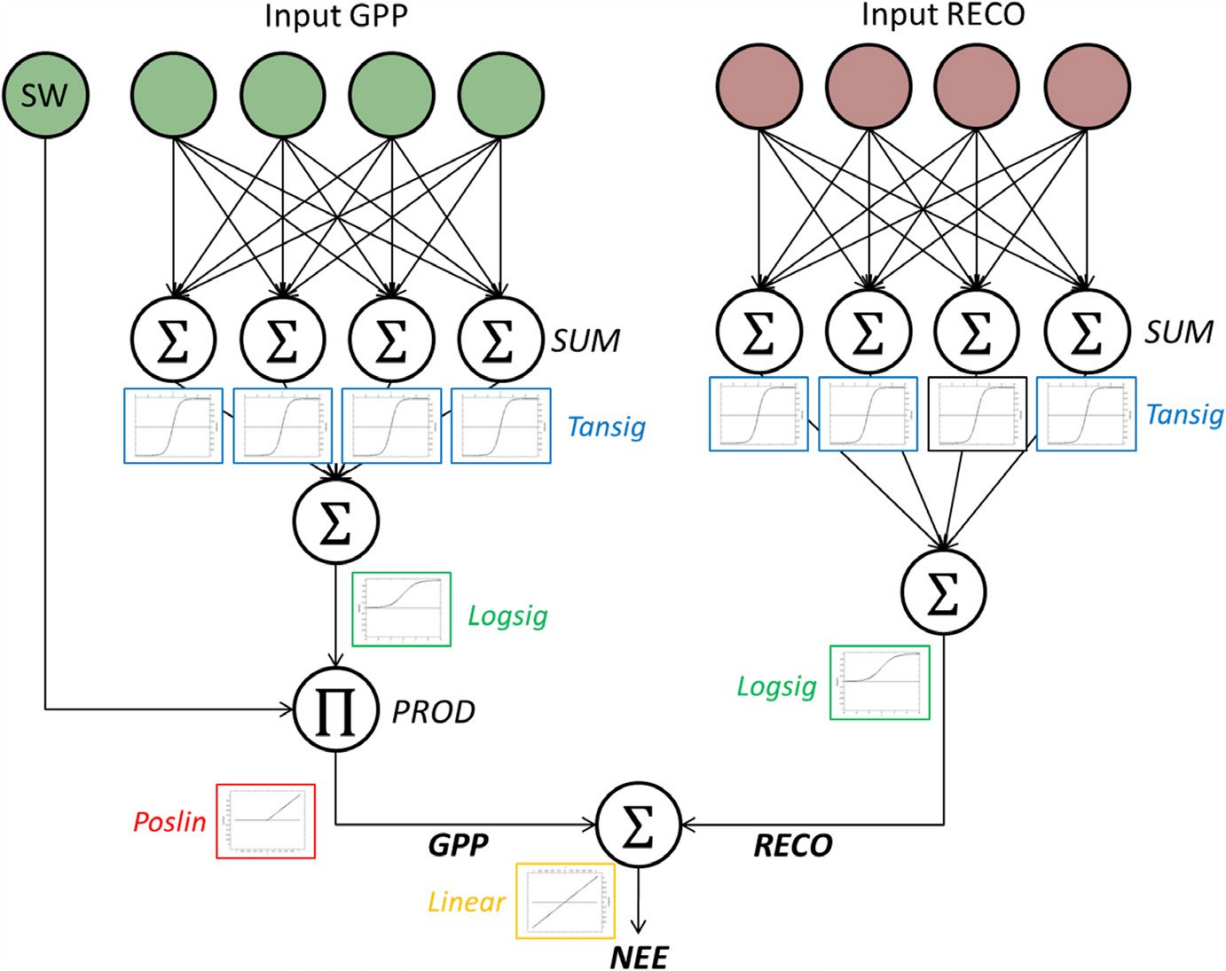
The scheme of the customized neural network applied for retrieving gross primary production (GPP) and ecosystem respiration (RECO) from net ecosystem exchange (NEE) measurements. The two subnetworks (SNN_GPP_ left, in green, SNN_RECO_ right, in brown) are connected in the last step to estimate NEE. Inputs for RECO are air and soil temperature; soil water content; wind speed and wind direction; the day of the year (sine and cosine values of angular day of year); the average value of nighttime NEE. Input GPP are, in addition to the shortwave incoming radiation used in the product, air temperature; vapor pressure deficit; soil water content; potential and actual shortwave incoming radiation; wind speed and wind direction; a proxy of the mean seasonal GPP dynamic derived from the nighttime and daytime averaged NEE. See Section 2.3 of the main text for details

In this experiment, we developed a new ANN architecture designed to emulate^1^The use of this term is not incidental: surrogate modeling, also known as emulation, is about developing statistical models that learn to mimic costly physical models, such as radiative transfer or climate models, using a representative dataset of simulation/forward runs. ML algorithms learn such parameter–observation relation with high accuracy, and after training can be used for forward simulation very efficiently (Bastos and O’Hagan, 2009; Camps‐Valls et al, 2019; Rivera et al., 2015; Vicent, et al 2018). the ecosystems processes driving NEE. This customized neural network (hereafter NN_C‐part_) is based on the concept that NEE, measured by the EC system, is the difference between RECO and GPP (Equation 1):

(1) NEE=RECO-GPP,

and that these two fluxes have individual drivers and dependencies. The uniqueness of our approach consists of imposing physical constraints in the proposed neural network, on the basis of the known properties of RECO and GPP. The overall structure is based on two subnetworks (Figure 1): one for retrieving RECO (sub neural network, SNN_RECO_) and the other for retrieving GPP (SNN_GPP_). The output of these two subnetworks (S_RECO_ and S_GPP_ for SNN_RECO_ and SNN_GPP,_ respectively) is combined in the last neuron of the overall structure, where NEE is calculated and compared with the measurements in order to optimize the network's weights (network training). The output signals of both subnetworks are constrained to be always positive, except for SNN_GPP_, which is constrained to be 0 during nighttime, as photosynthesis requires light. However, RECO and GPP have opposite signs; therefore, the connection weight of RECO is fixed to be positive (*w*_RECO_ = 1) and that of GPP to be negative (*w*_GPP_ = −1), mirroring the sign convention of the measured NEE. Since the last transfer function is unbounded and linear, and the bias is fixed to 0, the last node effectively reproduces the equation of NEE as:

(2) $${\text{NEE}}_{\text{ANN}}={\text{RECO}}_{\text{ANN}}+{\text{GPP}}_{\text{ANN}},$$

where

(3) $${\text{RECO}}_{\text{ANN}}=S_{\text{RECO}}*w_{\text{RECO}},$$

and

(4) $${\text{GPP}}_{\text{ANN}}=S_{\text{GPP}}*w_{\text{GPP}}.$$

The two subnetworks have different input drivers but a similar structure including two hidden layers: the first hidden layer has *n* hidden neurons (different for the two fluxes) and a hyperbolic tangent sigmoid activation function (“Tansig”), which improved the performance of network optimization; the second has only one neuron and a log‐sigmoidal activation function (“Logsig”) that constrains the output to positive values, allowing the implementation of Equation (2) in the last node. The SNN_GPP_, used to simulate GPP, has also a third layer where the measured incoming shortwave radiation enters as input in a node where a product is applied. This last node has a positive linear transfer function (“Poslin”); thus, the output from the SNN_GPP_ is 0 at nighttime and positive during the day. With this structure, the last node of the SNN_GPP_ mimics the light‐use efficiency (LUE) approach: the output coming from the two previous hidden layers is an LUE that is then multiplied by the available shortwave radiation to estimate GPP. In this way, the drivers are used to estimate an instantaneous proxy of the LUE which takes into consideration seasonal and diurnal variability of carbon uptake, including photosynthesis saturation at high light, while shortwave radiation mainly defines the magnitude of the flux. This decreases the weight given to the incoming shortwave radiation in relation to other driving variables. Up to this step, the output of the two subnetworks (S_RECO_ and S_GPP_) have positive signs; the sign convention of the NEE equation is finally restored in the last neuron of the overall structure by associating a positively signed weight to S_RECO_ and a negatively signed weight to S_GPP_.

### 2.2 Dataset used

For the purpose of this experiment, we used data from the FLUXNET2015 dataset (http://fluxnet.fluxdata.org) and, as an additional comparison, a synthetic dataset generated by the MUlti‐layer Simulator of the Interactions between a vegetation Canopy and the Atmosphere (MuSICA) process‐based model (Ogée, Brunet, Loustau, Berbigier, & Delzon, 2003, see section S4).

FLUXNET2015 includes meteorological and EC measurements that were quality checked and processed with standard tools (Papale et al., 2006; Pastorello et al., 2020) and provided with per‐variable quality flags. For more details, see the webpage http://fluxnet.fluxdata.org/data/fluxnet2015‐dataset/.

Data used for training and validation of the neural network were taken from the “FULLSET” “TIER 1” collection. Among the sites available in this collection, we selected a subset of 36 study sites (listed in Table 1) on the basis of the data quality and data availability in order to ensure the best conditions for the partitioning methods comparison. Site‐years were selected if the percentage of meteorological gap‐filled data was less than 20% and the measured NEE covered at least 10% of both daytime and nighttime periods.

**TABLE 1:** List of the subset of FLUXNET2015 study sites used in this experiment for net ecosystem exchange partitioning. The study sites used also for NN_C‐part_ validation (see section S1) are marked with the symbol (*) in the “Validation” column. Selected study sites represent the following International Geosphere–Biosphere Programme (IGBP) vegetation classes: Evergreen Needleleaf Forest (ENF), Deciduous Broadleaf Forest (DBF), Mixed Forest, Evergreen Broadleaf Forest (EBF), Grassland (GRA), Cropland (CRO), Woody SAvanna (WSA), Closed SHrubland (CSH), and Open SHrubland (OSH). For accurate coordinates, please refer to the FLUXNET2015 website

ID	Site code	IGBP	Lat	Lon	Validation	Reference
1	AU‐Cpr	SAV	−34,00	140,59	*	Meyer, Kondrlovà, and Koerber (2015)
2	AU‐DaP	GRA	−14,06	131,32		Beringer et al. (2011)
3	AU‐Dry	SAV	−15,26	132,37		Cernusak, Hutley, Beringer, Holtum, and Turner (2011)
4	AU‐How	WSA	−12,49	131,15		Beringer, Hutley, Tapper, and Cernusak (2007)
5	AU‐Stp	GRA	−17,15	133,35	*	Beringer et al. (2011)
6	BE‐Lon	CRO	50,55	4,75		Moureaux, Debacq, Bodson, Heinesch, and Aubinet (2006)
7	BE‐Vie	MF	50,31	6,00		Aubinet et al. (2001)
8	CA‐Qfo	ENF	49,69	−74,34	*	Giasson, Coursolle, and Margolis (2006)
9	DE‐Geb	CRO	51,10	10,91	*	Anthoni et al. (2004)
10	DE‐Gri	GRA	50,95	13,51	*	Prescher, Grünwald, and Bernhofer (2010)
11	DE‐Kli	CRO	50,89	13,52	*	Prescher et al. (2010)
12	DE‐Obe	ENF	50,79	13,72	*	
13	DE‐Tha	ENF	50,96	13,57	*	Grünwald and Bernhofer (2007)
14	DK‐Sor	DBF	55,49	11,64	*	Pilegaard, Ibrom, Courtney, Hummelshøj, and Jensen (2011)
15	FI‐Hyy	ENF	61,85	24,29	*	Suni et al. (2003)
16	FR‐LBr	ENF	44,72	−0,77		Berbigier, Bonnefond, and Mellmann (2001)
17	GF‐Guy	EBF	5,28	−52,92		Bonal et al. (2008)
18	IT‐BCi	CRO	40,52	14,96		Vitale, Di Tommasi, D’Urso, and Magliulo (2016)
19	IT‐Cp2	EBF	41,70	12,36	*	Fares, Savi, Muller, Matteucci, and Paoletti (2014)
20	IT‐Cpz	EBF	41,71	12,38	*	Garbulsky, Penuelas, Papale, and Filella (2008)
21	IT‐MBo	GRA	46,01	11,05	*	Marcolla et al. (2011)
22	IT‐Noe	CSH	40,61	8,15		Papale, Black, et al. (2015)
23	IT‐Ro1	DBF	42,41	11,93		Rey et al. (2002)
24	IT‐SRo	ENF	43,73	10,28	*	Chiesi et al. (2005)
25	NL‐Loo	ENF	52,17	5,74	*	Moors (2012)
26	RU‐Fyo	ENF	56,46	32,92	*	Kurbatova, Li, Varlagin, Xiao, and Vygodskaya (2008)
27	US‐ARM	CRO	36,61	−97,49		Fischer, Billesbach, Riley, Berry, and Torn (2007)
28	US‐GLE	ENF	41,37	−106,24	*	Frank, Massman, Ewers, Huckaby, and Negrón (2014)
29	US‐MMS	DBF	39,32	−86,41	*	Dragoni et al. (2011)
30	US‐NR1	ENF	40,03	−105,55	*	Monson et al. (2002)
31	US‐SRG	GRA	31,79	−110,83	*	Scott, Biederman, Hamerlynck, and Barron‐Gafford (2015)
32	US‐SRM	WSA	31,82	−110,87	*	Scott, Jenerette, Potts, and Huxman (2009)
33	US‐UMB	DBF	45,56	−84,71	*	Gough et al. (2013)
34	US‐Whs	OSH	31,74	−110,05	*	Scott et al. (2015)
35	US‐Wkg	GRA	31,74	−109,94		Scott, Hamerlynck, Jenerette, Moran, and Barron‐Gafford (2010)
36	ZA‐Kru	SAV	−25,02	31,50		Archibald et al. (2009)

Among the variables stored in the FLUXNET2015 dataset, we used the GPP and RECO obtained with the two standard methods: the nighttime method (NT) from Reichstein et al., 2005, and the daytime method (DT) from Lasslop et al., 2010. In the NT method, the Arrhenius‐type temperature–response curve of respiration as modeled by the Lloyd and Taylor equation (Lloyd & Taylor, 1994), driven by air temperature, is used for estimating RECO. This method makes use of the nighttime (when global incoming radiation <20 W/m^2^) NEE observations (assumed as representative of RECO given the absence of photosynthesis at night) to fit the Lloyd and Taylor equation. There are two parameters to fit in the Lloyd and Taylor equation: the activation energy (E0) and the respiration rate at the reference temperature (R_ref_). In the NT method, E0 is estimated at an annual scale by calculating E0 values every 15 days and then averaging the three with smaller uncertainty. Once E0 is fixed, the R_ref_ parameter is estimated using short‐term moving windows (4 days). On the basis of Equation (1), GPP is finally calculated by subtracting NEE from the daytime RECO extrapolated by the fitted model.

The DT method combines a rectangular hyperbola light response curve (Falge et al., 2001) for estimating GPP and the Lloyd and Taylor equation for estimating RECO (as in the NT method). In the DT method, nighttime NEE is used only to estimate the E0 parameter of the Lloyd and Taylor equation, while the remaining unknown parameters, including R_ref_, are fitted using daytime measurements. In DT, the light response curve is driven by the incoming shortwave radiation (SW_IN), but it is adjusted for the effect of stomata closure due to the atmospheric evaporative demand using VPD as an additional driver of GPP (Lasslop et al., 2010). Similar to NT: (a) the Lloyd and Taylor model is driven by air temperature, (b) the model parameters are fitted on short‐term moving time windows to account for slow changing factors.

### 2.3 Input variables and data preparation

The variable used as target values in the NN_C‐part_ training was the half hourly NEE (µmol CO_2_ m^−2^ s^−1^) measured with the EC technique. In particular, we used the NEE_CUT_USTAR50 variable (Pastorello et al., 2020). We used a comprehensive subset of micrometeorological variables measured at the flux towers as candidate drivers for NN_C‐part_, and additional variables derived from NEE, with the aim to explain variability of carbon fluxes at seasonal, daily, and half hourly resolutions. In particular, in terms of meteorological drivers, we used the SW_IN (W/m^2^), VPD (kPa), air temperature (TA [°C]), soil temperature (TS [°C]), and soil water content (SWC [%]) in the upper soil layer (depth function of the site, see Pastorello et al., 2020), wind speed (WS [m/s]), and wind direction (WD [degrees]). Each one of these variables is involved to different degrees in carbon exchange processes or in NEE measurements. SW_IN carries the photosynthetically active radiation that is a key variable for the light‐dependent reactions of photosynthesis, while leaves regulate stomatal conductance in response to VPD (Lasslop et al., 2010); temperature has a key role in chemical reactions of biological processes; thus, it is involved both in photosynthesis and in the RECO (Falge et al., 2001). SW_IN and VPD are used in the DT method to estimate photosynthesis while both DT and NT use TA to estimate RECO only. Wind‐related variables (WS and WD) affect the footprint of flux measurements (Arriga et al., 2017; Kljun, Calanca, Rotach, & W. and Schmid, H. P., 2015) which is important in particular if WD changes systematically (e.g., nighttime vs. daytime, morning vs. afternoon) and if the surrounding land cover is heterogeneous. The Julian day of the year (DOY) and the potential incoming radiation (SW_IN_POT [W/m^2^]) were also used in order to provide information about seasonality and length of the day. The SW_IN_POT was also aggregated at daily resolution to better track the seasonality of light conditions; the first derivatives of half hourly and daily SW_IN_POT were also calculated to add specific information about the seasonal and diurnal dynamics of light (Bodesheim, Jung, Gans, Mahecha, & Reichstein, 2018; Papale, Black, et al., 2015; Papale, Migliavacca, et al., 2015).

Finally, since plant photosynthesis and RECO change seasonally also due to substrate availability and management (in case of managed sites), daily average of nighttime NEE (NEE_NIGHT_) and a proxy of GPP (GPP_prox_) were calculated and used in input. In this case, gap‐filled (Reichstein et al., 2005) half hourly NEE was used in order to have a continuous time series. Proxy of GPP was calculated by using NEE_NIGHT_, and the average of daytime NEE (NEE_DAY_) as follow:

(5) $${\text{GPP}}_{\text{prox}}=\left({\text{NEE}}_{\text{DAY}}-{\text{NEE}}_{\text{NIGHT}}\right)*k,$$

where *k* is the fraction of daytime hours for each day.

Some of the drivers are periodic, which means that extreme values have similar meanings (e.g., the DOY 0 and 365 or the WD 0 and 360). To take this into consideration, we used their sine and cosine transformations instead of the original variable's value.

The two subnetworks that estimate RECO and GPP use a different set of drivers, selected on the basis of the expected role and tests on performances and that are listed in Table 2. In particular, SNN_RECO_ used TA, TS, SWC, WS, NEE_NIGHT_, and sine and cosine of WD and DOY while the drivers for the SNN_GPP_ were SW_IN, SW_IN_POT (half hourly and daily and their first derivatives), VPD, TA, SWC, WS, GPP_prox_, and sine and cosine values of WD. Note that SW_IN is then used both as driver and as single input in the GPP subnetwork for the product with the LUE (see Figure 1).

**TABLE 2:** List of the variables used as drivers to estimate gross primary production (GPP) and ecosystem respiration (RECO)

Variable	Time resolution	GPP	RECO
Measured	Half hourly	SW_IN^a^	TA
		SW_IN_POT	TS
		VPD	SWC
		TA	WS
		SWC	
		WS	
Calculated	Half hourly	SW_IN_POT First derivative	Cos WD
		Cos WD	Sin WD
		Sin WD	
	Daily	Daily average SW_IN_POT	Sin(DOY)
		Daily average SW_IN_POT first derivative	Cos(DOY)
		GPP prox	Net ecosystem exchange night

^a^ SW_IN is then used both as driver and as single input in the GPP subnetwork for the product with the LUE.

### 2.4 ANN training

Because our goal is to partition NEE into its two fluxes components, the NN_C‐part_ was trained at the site level and year‐by‐year; we used only high quality measured half hourly NEE (see Section 2.2) as training target. The training of the NN_C‐part_ was carried out using both nighttime and daytime measurements of NEE.

As commonly used in ANN training and application, inputs and outputs were normalized in the range +1/−1 by Equation (6):

(6) $$X_{\text{norm}}=2*\left[\left(\frac{X-X_{\min}}{X_{\max}-X_{\min}}\right)-0.5\right],$$

where *X*_min_ and *X*_max_ are estimated as the maximum of the absolute value of *X* then negative signed in the case of *X*_min_ and positive signed in the case of *X*_max_; this preserved the zero value in both normalized and original values without affecting the linearity of relationships between the original and the transformed time series.

Available records with all the inputs and the measured output were randomly split 25 times in sets for training (60% of the observations and, in general, at least 2,000 examples), test (20%) to avoid overfitting, and validation (20%) to assess the performances. This results in 25 datasets used for the ANN training. The ANN training used the mean square error as the cost function and the Levenberg–Marquardt backpropagation algorithm.

Five ANNs with different numbers of neurons in the first hidden layer of both subnetworks were trained: 10–13, 11–14, 12–15, 13–16, and 14–17 for SNN_RECO_ and SNN_GPP_, respectively. For each one of the 25 training datasets, the five networks with different structures were trained five times, changing the initial weights randomly, and then selecting the one with the best performance on the validation set. In case the sign of RECO and GPP did not respect the expected convention, the weights were reinitialized and the training was repeated. At the end of the process, there were 125 trained ANNs for each site‐year (25 training datasets × 5 structures). To select the best among them, which were then used for the application, the ANN with the highest performances on the validation set was selected for each training dataset (obtaining 25 selected ANNs), and then, the five ANNs with the best model efficiency in predicting the overall NEE time series were finally selected and applied. RECO and GPP were calculated by averaging the output of the two subnetworks SNN_GPP_ and SNN_RECO_ from the five selected ANNs. The capacity of the ANN to simulate NEE was also analyzed (see Data S1, section S1).

### 2.5 Statistical analysis and evaluation

Results of this experiment were evaluated by comparing GPP and RECO from NN_C‐part_ with the NT‐ and DT‐based partitioning methods in the FLUXNET2015 collection. As complementary information, we repeated the same analysis for the gross CO_2_ fluxes derived from the synthetic NEE dataset provided by the MUSICA model (hereafter, we explicitly refer the synthetic CO_2_ fluxes modeled with MUSICA as GPP_MUSICA_, RECO_MUSICA_, and NEE_MUSICA_). The GPP and RECO estimates from the different methods were compared at half hourly, daily, and yearly time resolutions and were further evaluated about the consistency among methods for the seasonal cycle and its daily anomalies. The seasonal cycle was estimated by averaging the fluxes over 5 days while the daily anomalies were estimated by subtracting the seasonal cycle from each daily value. Finally, in order to compare the seasonal cycles across sites‐years having different flux magnitudes, the annual average daily value was subtracted from each seasonal cycle. In addition to the seasonal cycles, the mean diurnal cycles were calculated and compared. To verify that the different drivers used in the NN_C‐part_ had the role that is ecologically meaningful, functional relationships between micrometeorological drivers and retrieved gross CO_2_ fluxes were analyzed. In particular, the functional relationship between SW_IN and GPP, VPD and GPP, and TA and both GPP and RECO were considered and analyzed. This was carried out in two different ways: (a) we simulated the flux responses in the NN_C‐part_ varying artificially one driver at time, while the other drivers were kept fixed to the average condition of the season at midday; and (b) we compared the seasonal variations of gross CO_2_ fluxes with respect to the meteorological forces for both fluxes calculated using the NN_C‐part_ results and those obtained using the NT and DT methods. The responses were obtained by averaging the values of 10 equal intervals step of the fluxes and drivers for 2 months.

The results of the machine learning approach were finally evaluated by looking at three specific responses of gross CO_2_ fluxes that are not explicitly accounted for by the DT and NT methods but can be indirectly considered in the NN_C‐part_ method: (a) the instantaneous LUE responses to the diffuse to direct radiation ratio, (b) the hysteresis of the diel cycles of predicted RECO and GPP with respect to TA and SW_IN, and (c) the respiration pulse after a rapid increase in SWC occurring frequently in dry environments. Finally, the NN_C‐part_ algorithm implemented here allows for an analysis of the role and importance of the different drivers (see Data S1).

## 3 RESULTS

### 3.1 Cross consistency between NN_C‐part_ and standard partitioning methods (DT and NT)

The half hourly GPP and RECO retrieved by NN_C‐part_ were consistent with the output obtained by the NT and DT methods, with a slightly higher correlation (*R* ^2^ and lower RMSE) with the DT method for GPP estimation and with the NT method for RECO, in both cases higher than the correlation between DT and NT methods (Table 3). The mean squared error of partitioned gross CO_2_ fluxes between NN_C‐part_ and the NT/DT methods was <1.53 µmol CO_2_ m^−2^ s^−1^ on average and comparable to the average value of the estimated random uncertainty of NEE (“NEE_CUT_USTAR50_RANDUNC,” variable provided by FLUXNET 2015). Looking at the bias, the magnitude of both GPP and RECO retrieved by NN_C‐part_ was slightly lower than the DT and NT methods, with the predicted values by NN_C‐part_ closer to the DT method (though the spread among methods was very small). The average values of bias among methods were low on average (<27 g C m^−2^ year^−1^) and comparatively lower with respect to the NEE random uncertainty reported in the FLUXNET2015 database (<10% of the reported “NEE_CUT_USTAR50_RANDUNC”). The agreement between NN_C‐part_ and the two FLUXNET standard methods was significantly lower in the case of RECO suggesting that the differences occurred in the diurnal and seasonal cycles of RECO between NN_C‐part_ and both DT and NT methods were comparatively larger than GPP.

**TABLE 3:** Cross consistency among the retrieved gross primary production (GPP) and ecosystem respiration (RECO) by NN_C‐part_, daytime method (DT), and nighttime method (NT) methods at half hourly time step, also after removing the mean daily value of the fluxes. Statistics reported in the table are the median of the study sites values, in brackets the 25th and 75th percentile

Gross CO_2_ flux	Variability	Comparison	Statistics		
			*R* ^2^	RMSE (µmol CO_2_ m^−2^ s^−1^)	Bias (µmol CO_2_ m^−2^ s^−1^)
GPP	Overall	NN_C‐part_ versus DT	.96 (.93/.97)	1.22 (0.76/1.52)	−0.042 (−0.17/0.039)
		NN_C‐part_ versus NT	.94 (.89/.95)	1.53 (0.89/2.02)	−0.068 (−0.18/0.025)
		DT versus NT	.90 (.84/.93)	1.84 (1.20/2.43)	0.020 (−0.16/0.14)
	Removing mean daily value	NN_C‐part_ versus DT	.95 (.93/.97)	0.93 (0.59/1.11)	
		NN_C‐part_ versus NT	.90 (.86/.92)	1.34 (0.82/1.86)	
		DT versus NT	.86 (.81/.89)	1.55 (1.02/2.13)	
RECO	Overall	NN_C‐part_ versus DT	.73 (.54/.83)	0.98 (0.70/1.29)	−0.023 (−0.19/0.12)
		NN_C‐part_ versus NT	.87 (.72/.92)	0.68 (0.47/0.83)	−0.072 (−0.16/0.022)
		DT versus NT	.78 (.65/.86)	0.95 (0.64/1.21)	−0.0045 (−0.21/0.16)
	Removing mean daily value	NN_C‐part_ versus DT	.21 (.09/.32)	0.39 (0.28/0.51)	
		NN_C‐part_ versus NT	.24 (.11/.38)	0.35 (0.26/0.47)	
		DT versus NT	.68 (.62/.76)	0.20 (0.14/0.26)	

**FIGURE 2:**
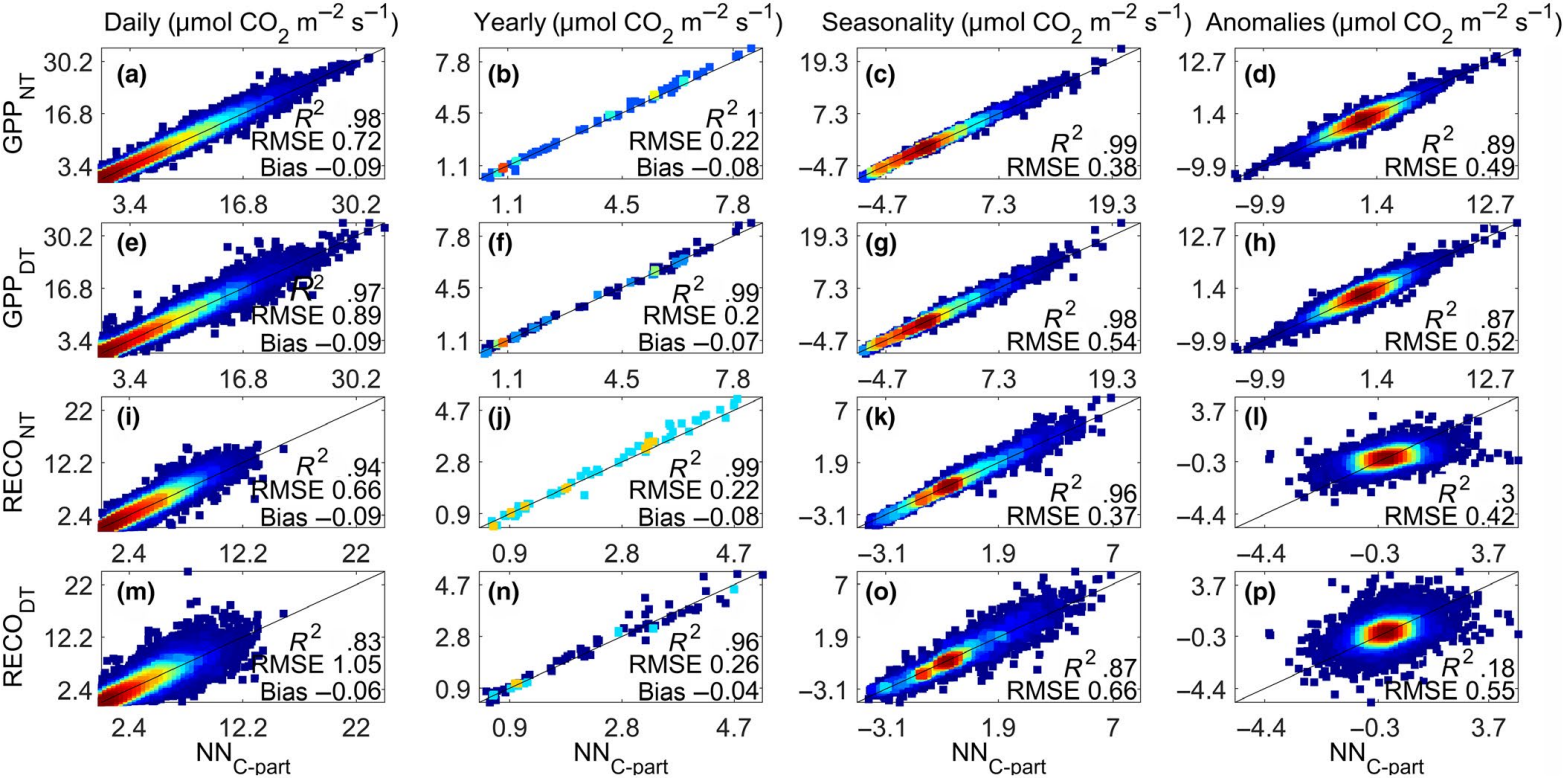
Scatter density plot showing the cross‐consistency between partitioned gross primary production and ecosystem respiration by NN_C‐part_ (*x*‐axis) and the ones by the daytime method and nighttime method methods (*y*‐axis) aggregated at daily time step (a, e, i, m), yearly (b, f, j, n),), and looking for the seasonal cycle (c, g, k, o) and daily anomalies (d, h, l, p)

High correlation and consistency among NN_C‐part_ and the DT and NT methods in both GPP and RECO were found for daily, seasonal, and yearly values and also for the daily average anomalies with respect to the seasonal values (Figure 2). Daily, growing season, and yearly average values from NN_C‐part_ were highly correlated with the two standard methods (*R* ^2^ > .83), in particular for GPP (*R* ^2^ > .97). In terms of bias, results by NN_C‐part_ were more consistent with the DT results confirming the findings from the subdaily comparison. High consistency was also found in the case of GPP daily anomalies (*R* ^2^ > .88 and RMSE < 0.52 µmol CO_2_ m^−2^ s^−1^), but it decreased in the case of RECO (Figure 2).

This general consistency between NN_C‐part_ and standard methods (DT and NT) was confirmed by comparing the gross CO_2_ fluxes derived by training the methods on synthetic NEE (NEE_MUSICA_, see section S4.1 in Data S1). The main findings from this comparison were that: (a) NN_C‐part_ and standard methods accurately retrieved both GPP_MUSICA_ and RECO_MUSICA_ at daily, yearly, seasonal timescales, and also the daily anomalies (see Figures S5 and S6); (b) NN_C‐part_ slightly outperformed the standard methods to estimate GPP_MUSICA_ and RECO_MUSICA_, particularly in the case of daily anomalies; (c) all methods slightly underestimated both GPP_MUSICA_ and RECO_MUSICA_ (bias ranging between −0.2 and −0.45 µmol CO_2_ m^−2^ s^−1^) with the largest discrepancy found in the case of DT method (for more details, see section S4.1, Figures S5 and S6).

### 3.2 Consistency among the seasonal and diurnal dynamics of partitioned gross CO_2_ fluxes

#### 3.2.1 Seasonal cycle

**FIGURE 3:**
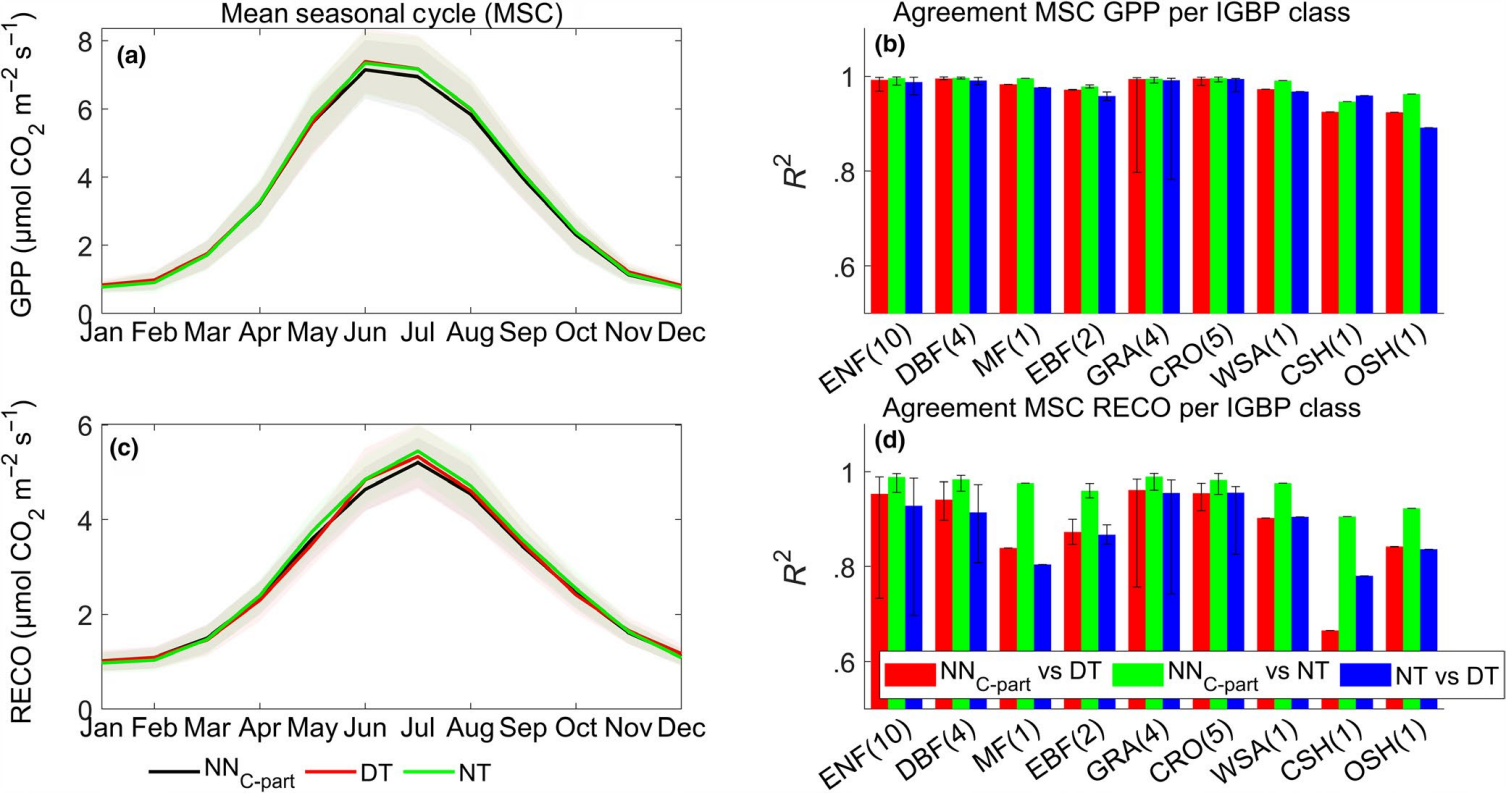
The mean seasonal cycles of gross primary production (GPP; a) and ecosystem respiration (RECO; c) obtained by NN_C‐part_, daytime method, and nighttime method. The consistency among methods was evaluated by the determination coefficients (*R* ^2^) among the seasonal patterns of GPP (b) and RECO (d) per International Geosphere–Biosphere Programme (IGBP) vegetation class (the number in brackets after each IGBP vegetation class stands for the number of sites in each category). Only FLUXNET2015 study sites at northern hemisphere (latitude > +15°N) were used for that analysis. The vegetation classes are the same as Table 1

The seasonal pattern of the retrieved gross CO_2_ fluxes by NN_C‐part_ closely matched that of the DT and NT methods, with the only exception being that predicted RECO by NN_C‐part_ was slightly lower in late spring/early summer (Figure 3, left). The predicted seasonal patterns were also similar among the represented International Geosphere–Biosphere Programme vegetation classes (Figure 3, right). Results showed a large degree of agreement among the three methods, in particular between NN_C‐part_ and NT.

The seasonal dynamic of gross CO_2_ fluxes derived from NEE_MUSICA_ by NN_C‐part_, NT, and DT agreed consistently. However, all methods underestimated both GPP_MUSICA_ and RECO_MUSICA_ (in particular the DT method) during the growing season (between April and July, see section S4.2 and Figure S7 in Data S1).

#### 3.2.2 Mean diurnal cycle of GPP

**FIGURE 4:**
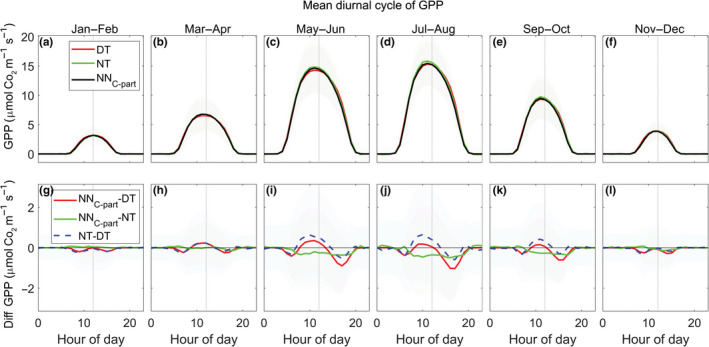
The trends of the mean diurnal cycle of gross primary production (GPP; a–f) retrieved by NN_C‐part_ (black line), daytime method (DT; red line), and nighttime method (NT; green line). The differences between diurnal cycle of retrieved GPP are also shown (g–l): NN_C‐part_‐DT (red line), NN_C‐part_‐NT (green line), and NT‐DT (dashed blue line). Only FLUXNET2015 study sites at northern hemisphere (latitude > +15°N), having at least 2 years of data, were used for the analysis

We found a good agreement between the pattern of the mean diurnal cycle of GPP predicted by NN_C‐part_ and FLUXNET standard methods (Figure 4). The GPP diurnal dynamic followed an asymmetrically bell‐shaped curve with the maximum value reached before midday (Figure 4, top panels). Analyzing the differences more in depth, the mean diurnal cycle of GPP predicted by NN_C‐part_ systematically diverged from the one predicted by the DT standard method, following a pattern that was consistent across the seasons (Figure 4, bottom panels). In particular, the GPP estimated by NN_C‐part_ was lower during the first hours of the morning (just after the dawn) and in the late afternoon; instead, the GPP estimated by NN_C‐part_ was slightly higher than DT from the morning until midday, where NN_C‐part_ estimated higher GPP values. A similar pattern appeared when comparing the two standard methods (difference calculated as NT‐DT) although shifted toward positive values, indicating higher values of GPP predicted by the NT method compared to the NN_C‐part_. The GPP derived from NEE_MUSICA_ confirmed the differences we highlighted between DT and the other methods (see Section 4.3; Figure S8).

#### 3.2.3 Mean diurnal cycle of RECO

**FIGURE 5:**
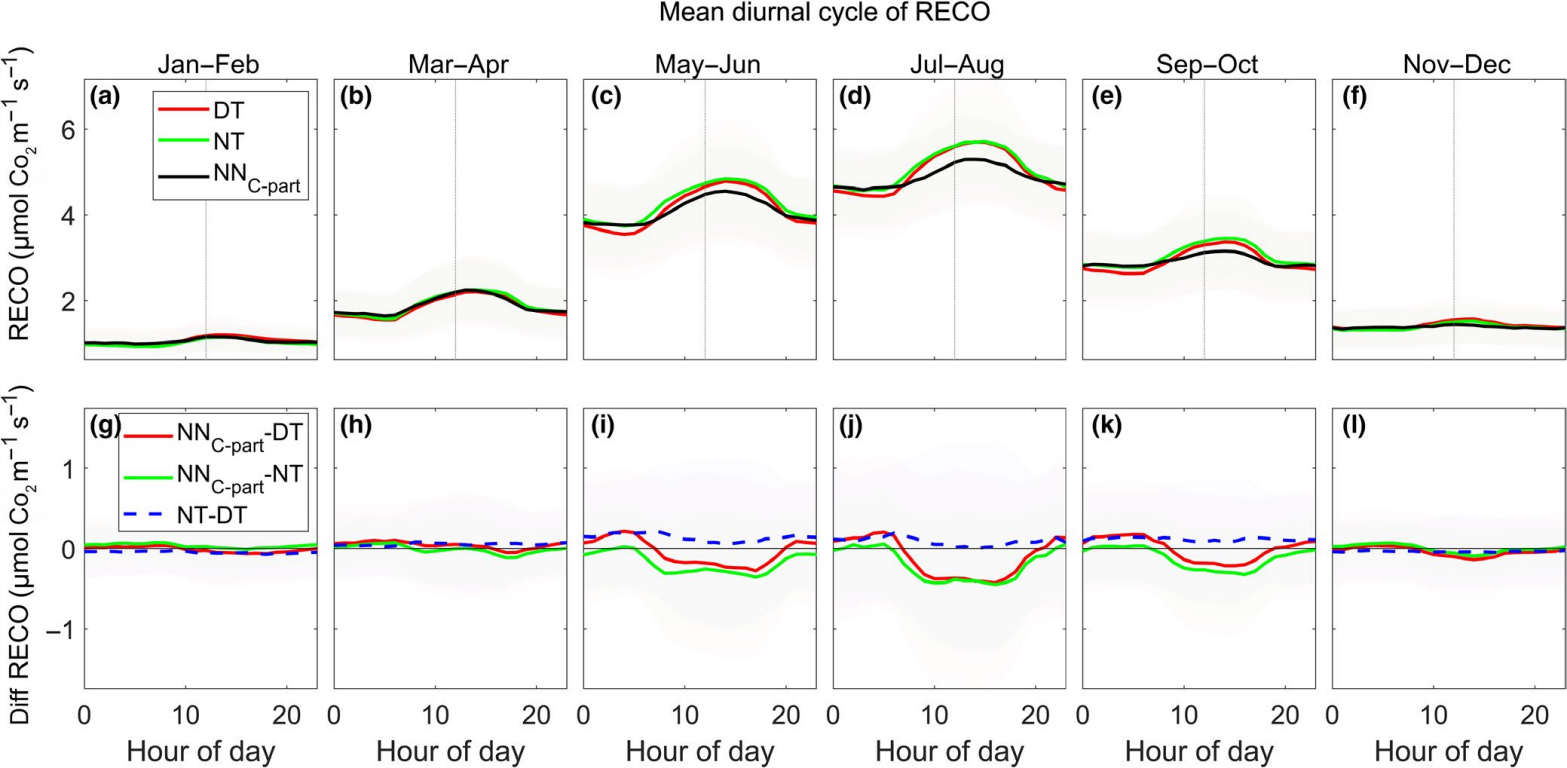
The trends of the mean diurnal cycle of ecosystem respiration (RECO; a–f) retrieved by NN_C‐part_ (black line), daytime method (DT; gray line), and nighttime method (NT; dashed gray line). The differences between diurnal cycle of retrieved RECO (g–l) are also shown: NN_C‐part_‐DT (continuous black line), NN_C‐part_‐NT (dashed black line), and NT‐DT (gray line). Only FLUXNET2015 study sites at northern hemisphere (latitude > +15°N), having at least 2 years of data, were used for that analysis

In comparison to GPP, the diurnal cycle of RECO modeled by NN_C‐part_ showed larger differences with respect to the DT and NT methods, in particular regarding the magnitude of the flux. All the methods showed an increased respiration when moving toward midday, in particular during the growing season (Figure 5, top plots); nevertheless, the NN_C‐part_ estimated values were lower than predictions from the other methods. Conversely, the differences between the two standard methods were negligible with a slightly higher RECO by NT in comparison to DT (Figure 5, lower plots, calculated as NT‐DT).

This finding was not fully mirrored in the patterns of RECO_MUSICA_ (see section S4.3 and Figure S9). In that case, NN_C‐part_ predicted higher RECO in comparison to both DT and NT, but more consistent with RECO_MUSICA_. Conversely, NN_C‐part_ predicted lower RECO than the NT method (and more consistent with DT) during the morning; the differences between DT and NT fitted on NEE_MUSICA_ were also enhanced. These differences increased during the nighttime hours, while the predicted RECO by the two standard methods became closer during the daytime hours (particularly when air temperature reached its maximum value). It is interesting to note that the peak of the maximum RECO by NN_C‐part_ was shifted compared to the DT and NT methods and closer to the one of RECO_MUSICA_, suggesting a better capacity to reproduce diurnal patterns when multiple drivers were involved (Figure S9).

### 3.3 Functional relationships between partitioned fluxes and meteorological drivers

**FIGURE 6:**
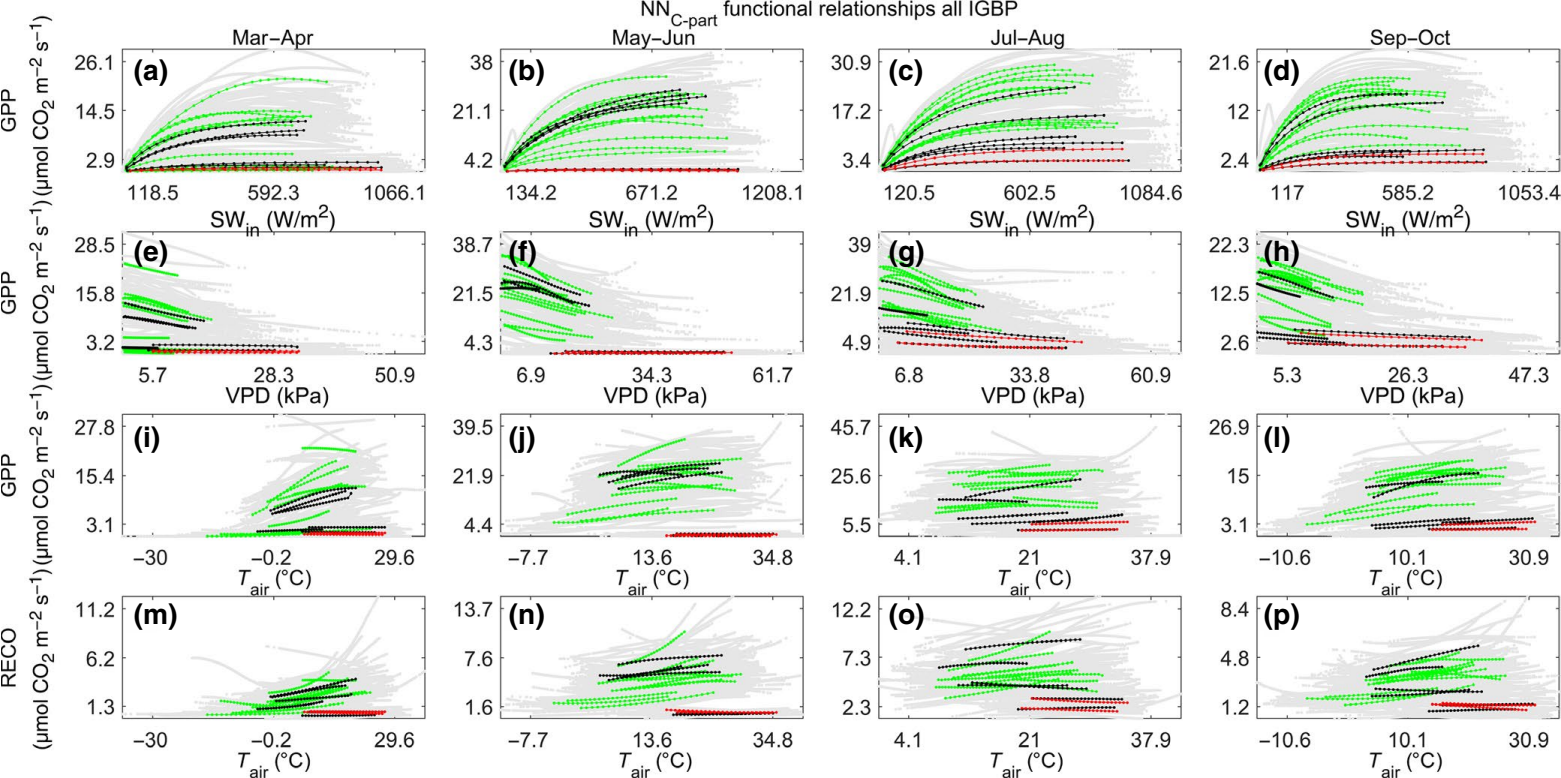
NN_C‐part_ predicted responses of gross primary production as function of SW_IN ([W/m^2^], a–d), VPD, ([kPa], e–h), Ta ([°C], i–l), and of ecosystem respiration as function of Ta ([°C], m–p). The functional responses are simulated in the study sites at the northern hemisphere (latitude > +15°N) and in fixed seasonal conditions. The average site‐specific responses (mean values of at least 3 years of data) are reported as colored lines, in particular: forested sites are in green, grassland and cropland sites are in black, and dry sites are reported in red. The singular site‐year response are dashed light gray lines

The functional response obtained by artificially varying the drivers in NN_C‐part_ was consistent with the current knowledge. The response of GPP to SW_IN was as expected, with an increased photosynthesis due to the increased light that resulted as saturated at high radiation values (Figure 6a–d). The response to VPD clearly showed the effect of VPD limitation on photosynthesis (Körner, 1995) that was evident also at relatively low values of VPD (Figure 6e–h).

The response of photosynthesis to air temperature showed a strong and positive response at the start of the growing season that became flat in the summer period (Figure 6i–l). The functional relationships between RECO and TA followed the expected increase of RECO with TA (Figure 6m–p) except for the dry ecosystems (red points in Figure 6) where TA was not the main driving factor. These general patterns of relationships were consistent across the seasons and in the different vegetation types, despite the expected variability due to the ecosystem‐specific properties.

**FIGURE 7:**
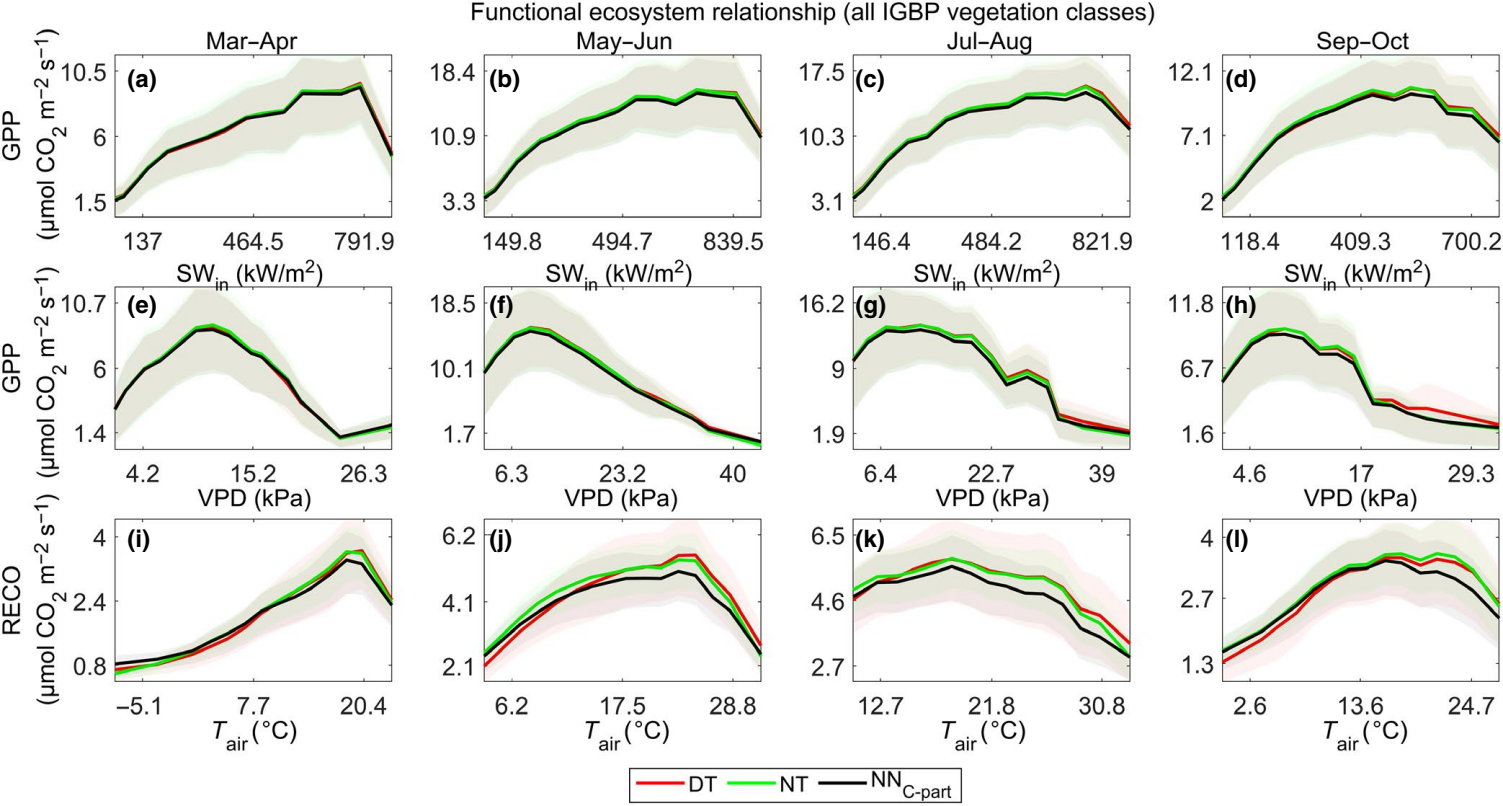
Univariate functional ecosystem relationships between gross CO_2_ fluxes retrieved by NN_C‐part_ (black lines) and FLUXNET standard methods (green and red lines for nighttime method and daytime method, respectively): gross primary production (GPP; µmol CO_2_ m^−2^ s^−1^) and SW_IN (W/m^2^; a–d), GPP (µmol CO_2_ m^−2^ s^−1^) and VPD (kPa; e–h), ecosystem respiration (µmol CO_2_ m^−2^ s^−1^) and TA (°C; i–l). These patterns are derived from study sites located at the northern hemisphere (latitude > +15°N)

When applied using the original measurements, the functional ecosystem response to the micrometeorological forces was largely consistent with the ones obtained using the DT and NT standard methods, although affected by multiple co‐acting and confounding factors (Figure 7). The shape of the relations was slightly different in the four periods analyzed, but it was important to recall that these were average responses from sites in different climatic conditions. It could be noted that an almost perfect match was present in the case of GPP at this broad scale; in the case of RECO, the NN_C‐part_ showed in general lower respiration fluxes at high temperatures (so mainly daytime) as opposed to the other methods, although the pattern of response was basically the same. This could be an indication of a water resources limitation during the summer period and at higher temperatures, that could be detected by the NN_C‐part_ since it also used SWC as a driver. In fact, the functional relationships between RECO and SWC retrieved by NN_C‐part_ highlighted a clear direct relationship between SWC and RECO in drought conditions, in particular for non‐forested ecosystems (Figure S16).

### 3.4 Additional ecological patterns simulated by NN_C‐part_

#### 3.4.1 NN_C‐part_ instantaneous LUE and the ratio between diffuse to direct radiation

**FIGURE 8:**
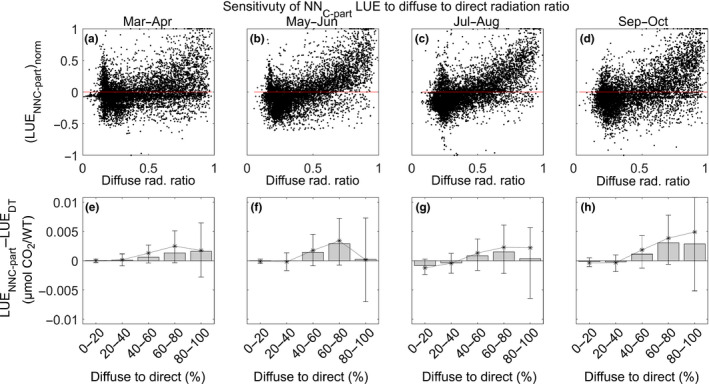
Sensitivity of the light use efficiency (LUE) of NN_C‐part_ to the diffuse to direct radiation ratio. The trend of LUE by NN_C‐part_ with respect to the diffuse radiation ratio is reported in the panels (a–d; here LUE is normalized for comparison purpose). The difference between LUE (µmol CO_2_/W) as estimated by NN_C‐part_ and that from the daytime method (DT) standard partitioning method is also reported (e–h); the statistics were aggregated per diffuse to direct radiation class (here estimated by the proxy 1‐SW_IN/SW_POT and reported as percentage). We reported the following statistics of *x* (*x* = LUE(NN_C‐part_) − LUE(DT)): the median values (bar), the mean value (*), the interquartile range (by brackets). Only LUE at midday is used in this figure

The instantaneous LUE estimated by NN_C‐part_ showed an increasing trend with respect to the increased diffuse to direct radiation ratio (here estimated by the proxy 1‐SW_IN/SW_IN_POT; Figure 8a–d).

When compared against the LUE estimated by the DT method, NN_C‐part_ showed a moderate but consistent higher sensitivity to the fraction of diffuse to direct light with a higher LUE when the diffuse to direct radiation ratio was higher than 40% (Figure 8e–h).

#### 3.4.2 Hysteresis of the diel cycle of gross CO_2_ fluxes

**FIGURE 9:**
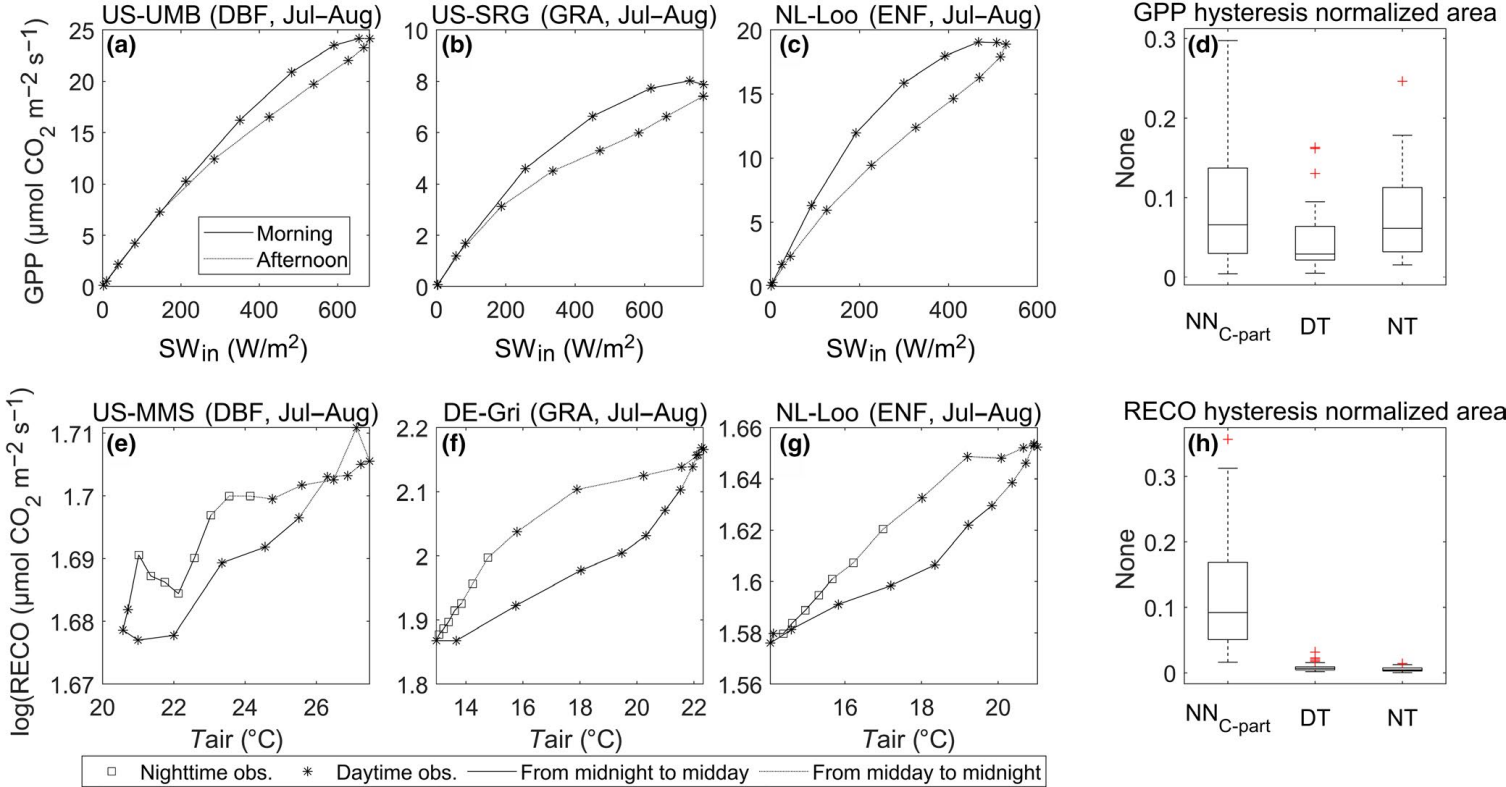
Hysteresis of the gross primary production (GPP) diel cycle predicted by NN_C‐part_ with respect to SW_IN in three sampled study sites (a–c). Three different International Geosphere–Biosphere Programme classes are reported: DBF (a), ENF (b), and GRA (c), while the period of interest was July–August. (d) Distribution of the GPP hysteresis integral area (then normalized) estimated by NN_C‐part_, nighttime method (NT), and daytime method (DT) partitioning methods. The hysteresis of the diel cycle of ecosystem respiration (RECO; here log‐transformed) with respect to the TA dynamic, in three sampled study sites, is reported (e–g). The distribution of the RECO hysteresis integral area (then normalized) estimated by NN_C‐part_, NT, and DT partitioning methods is reported (h)

The diurnal cycle of gross CO_2_ fluxes exhibited hysteresis, which we analyzed through the diurnal cycle of GPP with respect to SW_IN (Figure 9a–c) and the diurnal cycle of RECO with respect to TA (Figure 9e–g). We generally observed a clockwise cycle for GPP, with higher values of GPP in the morning in comparison to the afternoon. All the methods showed this pattern although it was more evident in the NN_C‐part_ and NT methods. We provided numerical estimates by calculating the integral area included in the hysteresis pattern (larger area indicates larger hysteresis) that was reported in Figure 9d.

For RECO, only NN_C‐part_ detected an appreciable hysteresis in the diurnal cycle (Figure 9h); this is expected because both the DT and NT methods use the same TA‐dependent and invariant relationships for the whole diel cycle. In general, the hysteresis of RECO is realized as a counterclockwise cycle, with the value of RECO, for the same TA, higher during the afternoon and nighttime hours and lower during the morning. However, the counterclockwise cycle observed in the RECO patterns was not always observed, and in some cases, the opposite hysteresis cycle was found.

#### 3.4.3 RECO pulses due to SWC variations

**FIGURE 10:**
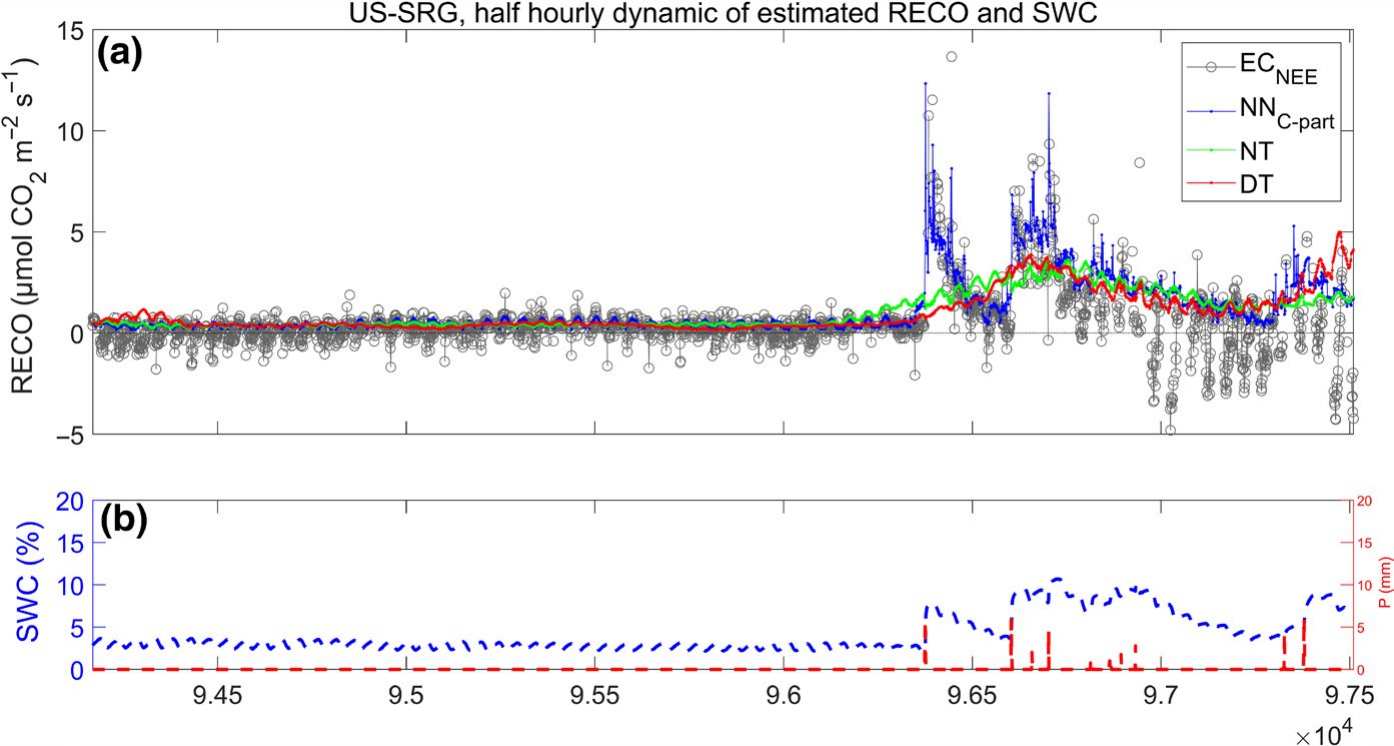
The sensitivity of half hourly ecosystem respiration (RECO) predicted by NN_C‐part_ to the SWC “pulse”. The investigated study site was US‐SRG. Fluxes are reported in panel (a) and more specifically we reported: RECO predicted by NN_C‐part_ (blue line), nighttime method (green) and daytime method (red) and the half hourly net ecosystem exchange (gray). Meteorological variables are reported in panel (b); more specifically we reported the SWC dynamics (blue line) and the rain events (*P* [mm]; red line)

The evaluation of the capacity to correctly reproduce rapid responses of RECO (respiration pulse) to changes in SWC (e.g., a post rain event after a prolonged drought period in arid environments) is in general difficult due to the sporadic nature of the events and the noise associated with the measurements. We evaluated the performances of the three models in a specific event that was clearly visible in the NEE time series (US‐SRG site, Figure 10). The analysis of the predicted RECO shows that NN_C‐part_ was much more sensitive to the fast response of respiration pulses. The NT and DT methods, that did not have SWC as a direct input, showed a slow changing pattern with the peak of respiration significantly delayed compared to the measurements.

## 4 DISCUSSION

### 4.1 Consistency of NN_C‐part_ functional response with theory on plant physiology and with the DT and NT standard methods

The retrieved GPP and RECO as obtained from NN_C‐part_ were consistent with the estimates by the DT and NT methods both when trained directly on EC measurements and using synthetic data with NEE_MUSICA_ (Figure 2; Figures S3 and S4). This was valid also for the relationship between micrometeorological drivers and gross CO_2_ fluxes calculated by NN_C‐part_ and those implemented in the DT and NT partitioning methods (Figures 6 and 7).

The GPP response to light used by NN_C‐part_ is curvilinear and consistent with the pattern of that used in DT method (despite some small but systematic divergences). The pattern of the GPP–VPD relationship captured by NN_C‐part_ is consistent with current knowledge about the protective (physiological) mechanisms of stomata closure carried out by plants in response to the increase in atmospheric evaporative demands (Körner, 1995). There is also a general consistency of the GPP–VPD dynamics retrieved by NN_C‐part_ with those from the other methods (Figure 7) although the relationships include also the effect of other covarying factors, in particular incoming radiation. This stomatal regulation of photosynthesis is explicitly inserted in the formulation only in the DT approach. Interestingly, the NN_C‐part_ shows an effect of VPD also at very low values (Figures 6e–h and 7e–h), lower than the 10 hPa used as threshold in the DT method. The patterns of the TA–RECO relationships are also consistent across methods and in agreement with the expected trend due to the biochemical reactions involved in the organic matter respiration processes that find their optimal conditions at high (but not extreme) temperatures, if not limited by water availability.

The largest mismatch we found between NN_C‐part_ and the two standard methods of partitioning (NT and DT) is in late spring/early summer, with lower fluxes of daytime RECO predicted by NN_C‐part_ compared to both standard methods. When evaluated on the synthetic dataset, the mismatch between NN_C‐part_ and the standard methods was significantly lower except for the anomalies, which were better reproduced by NN_C‐part_. In addition, the estimates from NN_C‐part_ better matched the diurnal dynamics of RECO_MUSICA_ than the estimates from the standard methods (Figure S9); conversely the method that showed the highest mismatch with respect to RECO_MUSICA_ was the DT method. All the methods underestimated both the reference GPP_MUSICA_ and RECO_MUSICA_ (despite the differences were very tiny) without any relevant effect on NEE. This could be related to a certain limitation of these methods to infer the complex relationships of the MUSICA model with the reduced set of drivers used in this experiment, but we have also to consider that both NN_C‐part_ and standard methods of partitioning were trained on noisy NEE signals (see section S4 in Data S1 for details) while both GPP_MUSICA_ and RECO_MUSICA_ used for reference are noise‐free.

Recent papers highlighted possible limitations of the standard methods for partitioning stemming from the fact that they do not consider the inhibition of daytime leaf respiration (the Kok effect; Keenan et al., 2019; Wehr et al., 2016) that produces an overestimation of daytime RECO and GPP in the NT method (Wehr et al., 2016) and an underestimation of nighttime respiration (see Keenan et al., 2019) in DT. Although some of the differences between the RECO estimated by NN_C‐part_ and the other methods (see Figures 3 and 5 and Figure S9) are consistent with experimental data focused on this topic (e.g., Keenan et al., 2019; Wehr et al., 2016), it is not possible to demonstrate that the NN_C‐part_ method, as implemented in this experiment, is able to reproduce the light inhibition of leaf respiration. Moreover, the consistency between the DT and NT partitioned fluxes used as reference in this experiment, and the pattern emerged by other modeling experiences (see e.g., Jung et al., 2020, where DT and NT partitioned fluxes were globally upscaled) suggests that the Kok effect could have a minor relevance on biases of RECO and GPP estimates compared to other sources of NEE measurement uncertainties.

In terms of ecological responses, the NN_C‐part_ outputs showed a clear and interesting response to the diffuse/direct light with an increased LUE in diffuse conditions and with hysteresis of the diel cycle of GPP and RECO that are only partially shown by the standard methods. The latter showed also strong limitation in reproducing the respiration pulse while NN_C‐part_ showed this capacity, although tested only in one case. In summary, these findings highlight the strength of this approach that is able to reproduce these patterns even without being trained specifically for this.

### 4.2 The advantages of the proposed NN_C‐part_ approach

One relevant difference between NN_C‐part_ and the standard partitioning methods is the absence of prescribed relationships between drivers and fluxes. In fact, only a few constraints have been set in the NN_C‐part_ structure (RECO and GPP signals must be positive, weights in the last node have to be consistent with the sign convention of NEE) and the relationship between inputs and outputs is set on the basis of the data without any assumption (as common in all the machine learning methods). This property could be a great advantage when used to study a phenomenon that does not respond with the same invariant pattern. The synthetic datasets used in this experiment are generated using functional relationships which are more complex than the ones implemented in the FLUXNET standard methods. The better matching by NN_C‐part_ highlights the potential of this algorithm to capture additional patterns of carbon fluxes dynamics. As a direct consequence, this led to some systematic differences observed in the GPP and RECO patterns compared to standard methods. For instance, the shape of the GPP diurnal cycle predicted by the DT method is almost symmetrically bell shaped: GPP reached a maximum around midday and only VPD, used as a downregulation function, when higher than 10 hPa, can modify this pattern that is otherwise dictated by incoming radiation. This is also visible in the modeled NEE, by comparing NN_C‐part_ and DT outputs with measured data (Figure S10). Conversely, the pattern of GPP obtained by the NT method is less affected by prescribed relationships because GPP is calculated as the difference between RECO and the measured NEE. The diurnal dynamic of GPP estimated by NN_C‐part_ is more similar to the NT estimates, which suggests that the formulation of the DT approach could be improved, for example, by using the non‐rectangular light response function (instead of the rectangular used in DT, see Gilmanov et al., 2003) or a different threshold (or a different function) for the VPD downregulation effect. Although these differences did not lead to large mismatches in terms of magnitude and/or seasonal dynamics of GPP, they can have an effect on the estimates of ecosystem functional properties such as LUE, WUE, or other important physiological parameters (Keenan et al., 2019; Reichstein, Bahn, Mahecha, Kattge, & Baldocchi, 2014).

Machine learning algorithms, such as the one implemented in this experiment, are also more effective at extracting relevant features from the comprehensive set of drivers used as input. For example, NN_C‐part_ indirectly derived information on diffuse radiation from the measured and potential incoming radiation used as drivers to scale the LUE, as consistently found in other studies (Alton, North, & Los, 2007). In addition, NN_C‐part_ directly uses additional input to improve the estimates of GPP. For example: (a) TA shows a role in spring and then a less strong effect possibly due to acclimation (Figure 6i–l); (b) SWC could play a role in downregulating the GPP response to VPD, with the effect of the atmospheric evaporative demand being stronger in the case of reduced soil water availability from soil and effective water stress condition.

In the case of RECO, both DT and NT used the same modeling approach, based on the Lloyd and Taylor model driven by the air temperature, resulting in high consistency between their results. However, it is known that soil temperature is also an important driver of RECO in particular to define its temporal (diurnal) dynamic (Lasslop et al., 2012; Wohlfahrt & Galvagno, 2017) because it is directly linked to soil respiration, which is a large contribution to the total RECO (Misson et al., 2007). The NN_C‐part_ method uses soil temperature (in addition to air temperature) as a direct driver of RECO and the analysis of the functional responses highlighted an important and direct effect (Figure S3). By looking at the analysis of RECO driver importance (see section S8 and Figure S20 in Data S1), it seems that TS and TA had, in general, a similar importance, but their relative weights changed by site. For this reason, it could be difficult to use both TA and TS in the same Lloyd and Taylor relationship without a priori information about the relative importance of the two variables. The multiple drivers approach implemented in NN_C‐part_ where both TA and TS are used as drivers of RECO without any a priori assumptions also allows for the detection of hysteresis in the diurnal cycle of RECO (see Wohlfahrt & Galvagno, 2017 for details on the topic).

Another important driver of RECO that is not explicitly accounted for in the standard methods was SWC that is particularly important in dry sites (see section S8). The DT and NT methods use a dynamic parameter (R_ref_) estimated using moving windows to indirectly consider the slow changes in water availability (Reichstein et al., 2005). Soil water content, however, also has instantaneous effects in some ecosystems (e.g., the respiration pulses after short rain events, see Jarvis et al., 2007) that need the direct use of SWC as input, like in the NN_C‐part_ method, to be effectively modeled.

Other slow changing factors affecting the fluxes, such as phenological state, substrate availability, and management or other disturbance events, that are considered indirectly through R_ref_ in the standard methods, are represented in the NN_C‐part_ approach through the use of averaged NEE‐derived quantities as drivers.

Finally, the standard methods do not account for EC footprint variability, which can significantly affect the magnitude of the measured fluxes if the fetch is small and not fully homogeneous (see section S8). This could be considered, for example, by fitting the models per wind sectors and wind speed classes (or using footprint models), but the amount of available data could become critically low in less represented conditions. In the NN_C‐part_, we used wind variables (direction, speed) as input in order to indirectly take into consideration footprint variability. Wind‐related variables appeared to be important in a few study sites (see Figures S15, S16, and S17). By comparing the ANN trained by including/excluding wind variables, we detected an effect on performance (increase of RMSE), which highlights an important effect on the instantaneous estimated flux values. However, the role of wind variables is reduced in the yearly budget calculation of gross CO_2_ fluxes (~6 g C m^−2^ year^−1^, average value across study site) even though differences were detected site by site (site‐specific differences ranged between −169 and +122 g C m^−2^ year^−1^). In summary, the implemented machine learning approach gives the possibility to use variables as drivers which would be difficult to consider in process‐based approaches, which require that the role and effects of each single variable being known and correctly encoded (at the ecosystem scale).

### 4.3 Uncertainty and limitation of the proposed method

Despite the encouraging results, there are also limitations and uncertainty sources to consider when a pure machine learning approach is used. The absence of prescribed relationships and the few constraints fixed made the method robust against incorrect or incomplete formulations used in the standard methods but also led to higher sensitivity to the uncertainty in the data used in the training phase of machine learning, particularly when both NEE and the meteorological variables used as drivers are affected by long gaps. In the NN_C‐part_, the weights of the ANN are optimized against NEE; thus, the uncertainty of NEE could have a significant impact on their estimation. This could be particularly relevant for nighttime NEE measurements that can be affected by higher uncertainty (e.g., advection, large footprint) that, even if filtered, lead to long gaps. In these situations, a model based on ecological responses is in general more robust, and needs less data for a proper calibration than a data‐driven approach.

Another limitation is related to the availability of the large set of input data that this approach requires in order to fully exploit its advantages, which are often not fully available at all FLUXNET sites. In this situation, the model can be used with a reduced set of drivers, but this could also lead to higher uncertainty and lower performance in specific sites, while models based on ecological processes like the NT and DT may be more robust because the response pattern is already prescribed.

## 5 CONCLUSIONS

In this study, we propose a machine learning method for NEE partitioning (NN_C‐part_) using an ANN approach with a tailored structure to simultaneously retrieve GPP and RECO. From a methodological point of view, this approach is an example of simple hybrid modeling (Reichstein et al., 2019), combining a neural net with a “theoretical” ecosystem equation based on LUE.

The NN_C‐part_ is designed to use as input a comprehensive set of drivers selected among the most commonly measured variables at EC sites. This property of NN_C‐part_ (and of machine learning in general) can be exploited in future studies, for example, using as input other variables such as diffuse radiation, COS, ^13^C isotopes, or SIF. Additionally, NN_C‐part_ does not make use of prescribed relationships between inputs and outputs and only a few constraints are introduced. Results in terms of the consistency of the gross CO_2_ estimated fluxes by NN_C‐part_ with the estimates from FLUXNET standard methods were good and the proposed model is able to reproduce magnitudes, patterns, and functional responses. However, the systematic differences emerged by the cross‐consistency analysis and the ecological patterns captured by NN_C‐part_ suggest that the implemented ANN reproduces fluxes dynamics more realistic than standard methods of partitioning. Being that said, further investigation are required in order to clarify the impact of the additional drivers used in NN_C‐part_, or possible missing functions and relationships in the standard methods.

Altogether, this new method provides GPP and RECO estimates that are based on purely data‐driven empirical relationships without any assumption of the driver–output relations. This product would be of high interest for process model parameterization and validation as it avoids unwanted circularity, and for this reason, we propose it as a complement to the standard processing in large networks such ICOS, AmeriFlux, or FLUXNET.

## Supporting information

Data S1

## Data Availability

All the data used in the paper are included in the FLUXNET2015 collection available at https://fluxnet.fluxdata.org/ and shared under the CC4BY license. The codes for preprocessing and preparing data for partitioning, train the algorithm, select the best ANN, and produce the outputs are also shared under the BSD‐Clause3 open license in GitHub at the address https://github.com/icos‐etc/Partitioning_ANN.
