# Supplementary material for: Partitioning net carbon dioxide fluxes into photosynthesis and respiration using neural networks
Source: Glob Chang Biol. 2020 Jul 2;26(9):5235–53. doi: 10.1111/gcb.15203 (PMC7496462; doi:10.1111/gcb.15203)
Supplement: Supplementary file 1 — Data S1 [file GCB-26-5235-s001.docx]

**Supplementary information**

**S1 Validation of NN_C-part_ on NEE.**

As an additional test for evaluating the generalization capability of the trained ANN, we carried out a cross-validation experiment on the FLUXNET study site used for the partitioning experiment. The overall training processes, from the preprocessing of input data to the models parameters tuning, was the same used in the partitioning application (see section 2.4 of the main text for details). Only sites with sufficient data coverage to allow artificial gaps of 30% were selected and this resulted in a total of 23 sites (see main text, Table 1).

The validation done on a subset of 30% of data not used in the training show a high R^2^ and reduced RMSE and BIAS, with a high level of consistency across the sites used (Table 3). The model performances have also been analyzed on the daytime and nighttime fluxes separately showing higher performances during daytime, as partially expected due to the potential errors (random and systematic) that in general affect more the nighttime data.

Table S1: Statistics from cross-validation of NN_C-part_ on NEE measurements. Performances reported for the overall data and also for the daytime and nighttime data separately. The median value across the study sites is reported, in brackets the 25^th^ and 75^th^ percentile.

| Nighttime/Daytime Data | Statistics | | |
| --- | --- | --- | --- |
|  | R^2^ | RMSE [µmol CO_2_ m^-2^ s^-1^] | BIAS [µmol CO_2_ m^-2^ s^-1^] |
| Overall | 0.91 (0.87/0.93) | 1.53 (0.97/2.17) | 0.0114 (-0.0174/0.0397) |
| Daytime | 0.90 (0.86/0.92) | 1.74 (1.12/2.49) | 0.0124 (-0.0293/0.0719) |
| Nighttime | 0.67 (0.53/0.73) | 1.04 (0.68/1.45) | -0.017 (-0.0697/0.0285) |


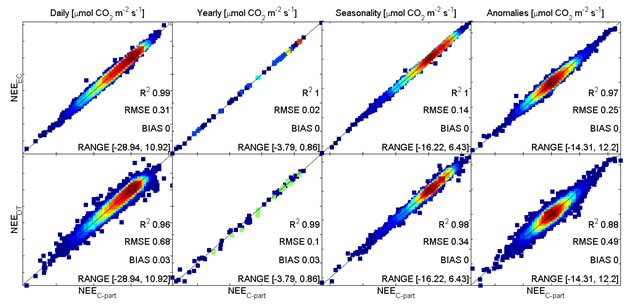


Figure S1: the fitting of NEE estimated by NN_C-part_ as compared with NEE measured by EC towers and with the estimates by DT standard partitioning method.

**S2 Cross correlation between yearly values of GPP and RECO predicted by NN_C-part_, NT and DT for the FLUXNET study site used in this study.**


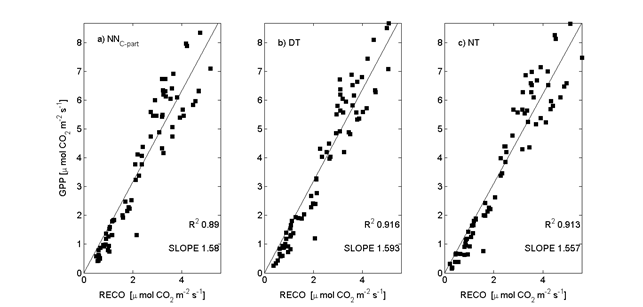


Figure S2: between site cross-comparison mean annual GPP vs mean annual RECO

**S3 Scaling of the uncertainty of partitioned fluxes compared to NEE random uncertainty**

**
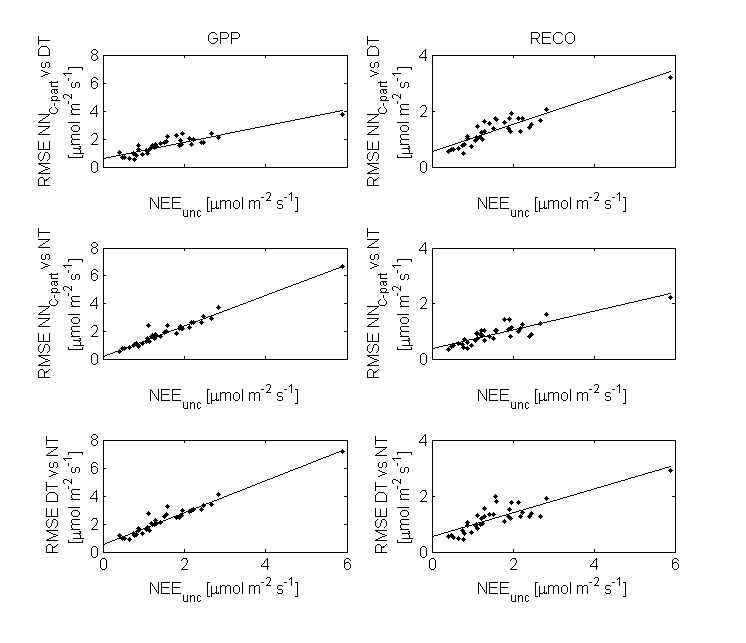
**

Figure S3: scaling of the standard error (RMSE) of GPP and RECO compared to the increasing NEE random uncertainty.


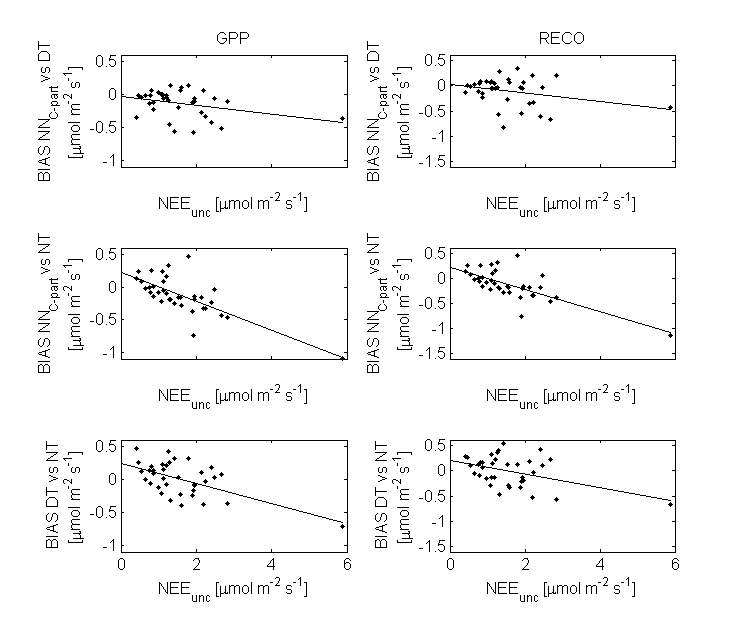


Figure S4: same as figure S3 but for BIAS.

**S4 Analysis on the synthetic dataset**

As an additional comparison we tested NN_C-part_ algorithm with an artificial dataset of NEE provided by the process based model MUSICA (Ogee et al., 2003). In this way we could rely on a reference where input and output relationships were known (despite the simplifications always introduced in a model). NN_C-part_, NT and DT methods were trained/optimized on NEE_MUSICA_ at which artificial noise were added. The artificial noise results by the sum of: a) a white Gaussian-noise with 0 mean and 0.1 standard deviation; b) an heteroscedastic random noise component that scale for the magnitude of the output (~ 7.5 % of the magnitude of NEE). The modeled study sites (year 2006-2008) were reported in Table S2.

Table S2: list of the study sites modeled by MUSICA. The synthetic NEE of the following sites were used to train NN_C-part_ and to fit the NT and DT partitioning method.

| ID | Site Code | Name | Country | IGBP | Latitude | Longitude | Elevation |
| --- | --- | --- | --- | --- | --- | --- | --- |
| 1 | DE-Tha | Tharandt | Germany | ENF | 50.9624 | 13.5652 | 385 |
| 2 | FI-Hyy | Hyytiala | Finland | ENF | 61.8474 | 24.2948 | 181 |
| 3 | FR-LBr | LeBray | France | ENF | 44.7171 | -0.7693 | 61 |
| 4 | FR-Hes | Hesse | France | DBF | 48.6742 | 7.0646 | 300 |
| 5 | DE-GR | Gruenschwaige | Germany | GRA | 48.38 | 11.83 | 500 |

For the training on the MUSICA dataset, we used the same variables used in the FLUXNET2015 experiment with the exception of WD because the latter is not available. However, for the purpose of the analysis carried out with the synthetic data and for the effect produced by WD on the partitioned fluxes, it did not affect the findings of this experiment.

*S4.1 Cross consistency of modeled gross CO_2_ fluxes at daily, yearly seasonal time scale, including the daily anomalies.*

The outputs from NN_C-part_, NT and DT were compared with GPP_MUSICA_ and RECO_MUSICA_. NN_C-part_ generally (slightly) outperformed the standard methods of partitioning, in particular the estimates of GPP anomalies. In general for NN_C-part_, the R^2^ ranged between 0.99 and 0.93, while RMSE between 0.53 and 0.26 µmol CO_2_ m^-2^ s^-1^. The performance of standard methods (DT and NT) were slightly lower. About GPP_MUSICA_, the R^2^ ranged between 0.98 and 0.86 and the RMSE between 0.79 and 0.26 µmol CO_2_ m^-2^ s^-1^, see Figure S5).


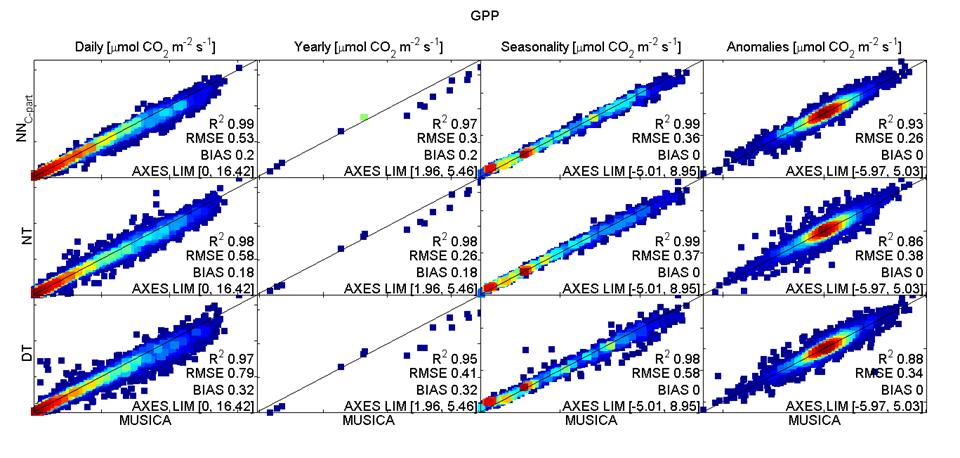


Figure S5: cross comparison of GPP (aggregated) estimated by NN_C-part_, NT and DT methods on the synthetic NEE_MUSICA_. Here GPP_MUSICA_ was used for comparison and reported in the x-axis, while GPP from the other methods are on the y-axes. BIAS is calculated as the difference x-y. As in Figure 2 of the main text, we evaluated the yearly and daily aggregated fluxes, the mean seasonal values (seasonality), and the daily anomalies.

Similar results were also found in the case of RECO_MUSICA_, despite a general lower R^2^, and higher RMSE, for all the methods (Figure S6).


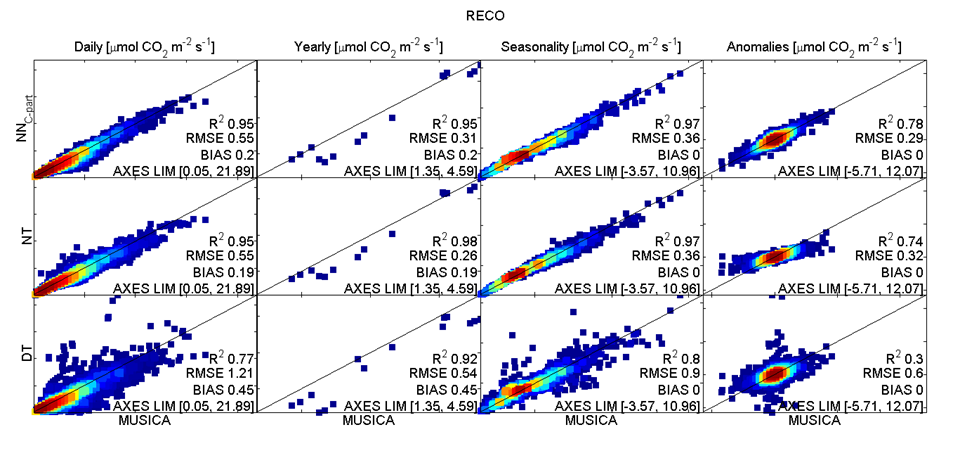


Figure S6: same as in Figure S5 but for comparison with RECO_MUSICA_.

In terms of BIAS, NN_C-part_, NT and DT underestimated both GPP_MUSICA_ and RECO_MUSICA_. The methods with the highest BIAS (for both GPP_MUSICA_ and RECO_MUSICA_) was the DT being the mean difference between the estimated values and the gross CO_2_ fluxes from MUSICA (here calculated as MUSICA-DT) roughly 0.32 and 0.45 µmol CO_2_ m^-2^ s^-1^ for GPP and RECO respectively (almost two times the BIAS of NT and NN_C-part_).

*S4.2 Cross consistency of the mean seasonal cycle of modeled gross CO2 fluxes.*


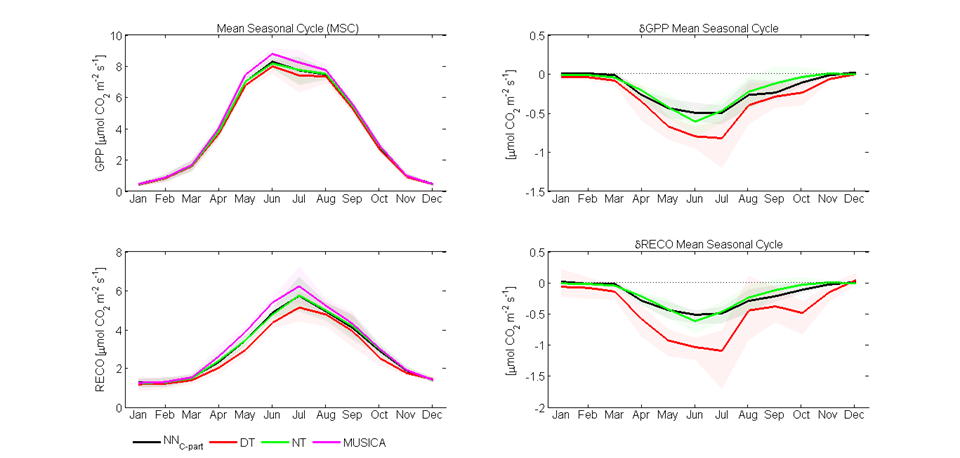


Figure S7: On the left, the comparison among the mean seasonal cycle of GPP (top) and RECO (bottom) predicted by NN_C-part_, NT and DT, fitted on NEE_MUSICA_; the reference GPP_MUSICA_ and RECO_MUSICA_ are reported in magenta. On the right, the differences between NN_C-part_, NT, DT and the references from MUSICA (GPP_MUSICA_ and RECO_MUSICA_).

*S4.3 Cross consistency of the mean diel cycle of modeled gross CO_2_ fluxes.*

The estimates of the mean diurnal cycle of GPP by NN_C-part_, DT and NT methods showed a good agreement with GPP_MUSICA_ despite all methods seems slightly underestimating the maximum value of GPP (Figure S8)**.**


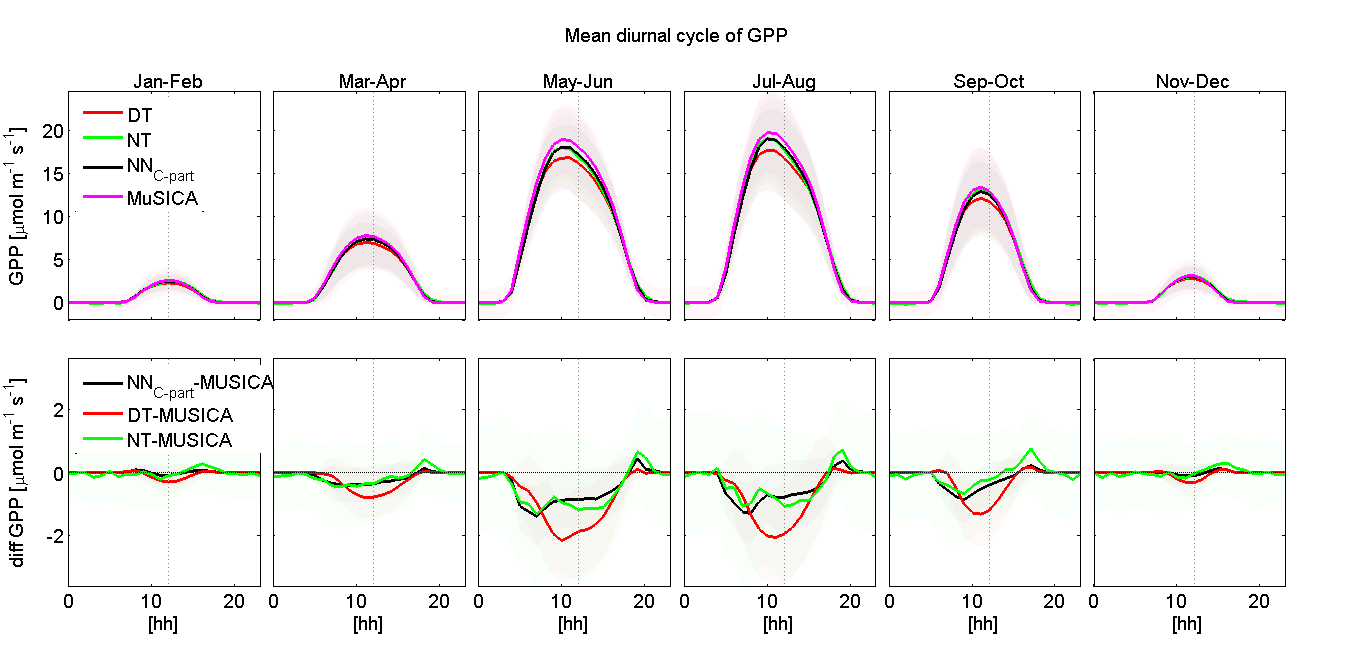


Figure S8: top panels, the dynamic of the mean diel cycle of GPP predicted by NN_C-part_, DT and NT standard methods trained on the NEE_MUSICA_. GPP_MUSICA_ was reported as reference. Bottom panels, the difference between GPP from NN_C-part_, DT and NT and the reference from GPP_MUSICA_.

The estimates of the mean diel cycle of RECO by NN_C-part_, DT and NT methods showed a lower agreement with RECO_MUSICA_ compared to GPP; moreover all methods underestimate the maximum value of RECO (Figure S9)**.**


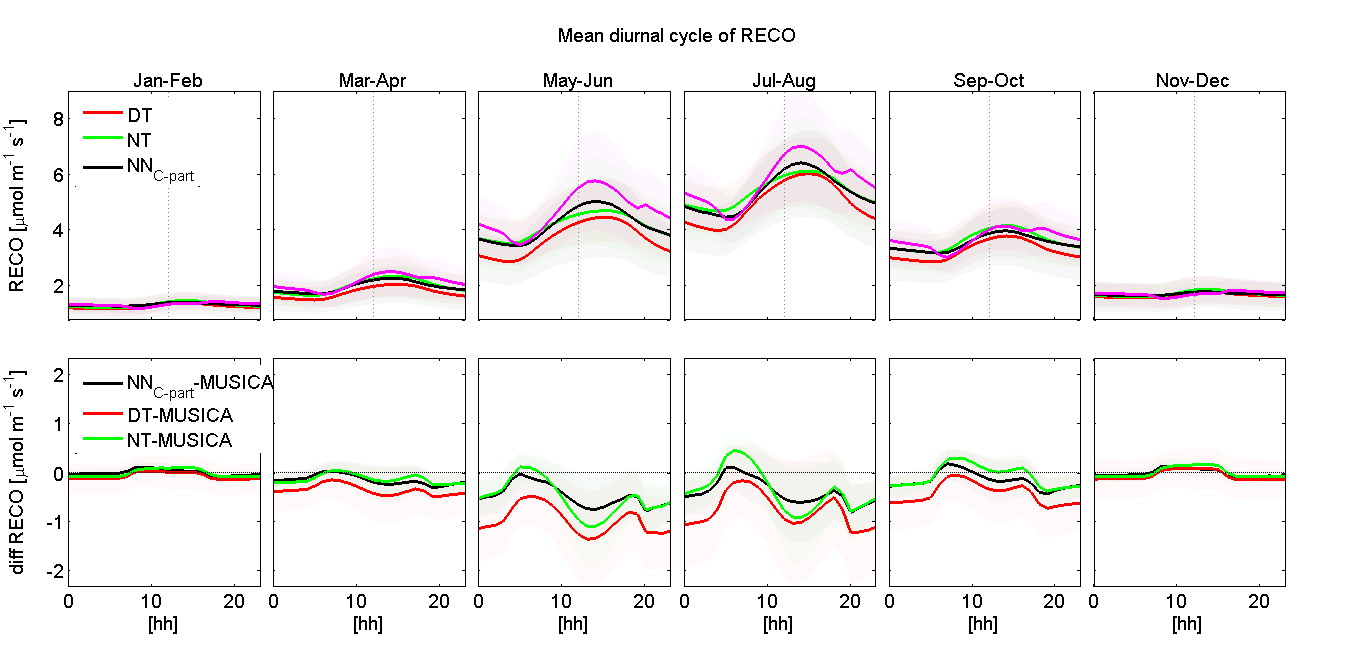


Figure S9: as in Figure S8 but for RECO.

**S5 Comparison among the diurnal cycle of measured NEE and as estimated by NN_C-part_ and DT methods.**

In this section we compared the mean diurnal cycle of NEE fitted by NN_C-part_, the eddy covariance NEE measurements and the NEE fitted by the DT standard method (see supplementary information). The pattern shows that DT method underestimates NEE in early morning and afternoon and overestimate NEE during the central part of the day; this is consistent with possible overestimation (underestimation) of GPP by DT during the early morning and afternoon (during the central part of the day).

An almost perfect match was found comparing NEE fitted by NN_C-part_ and the eddy covariance measurements; however it is important to remind that the NN_C-part_ method is fitted using all the EC measurements while the DT method used only the daytime data.


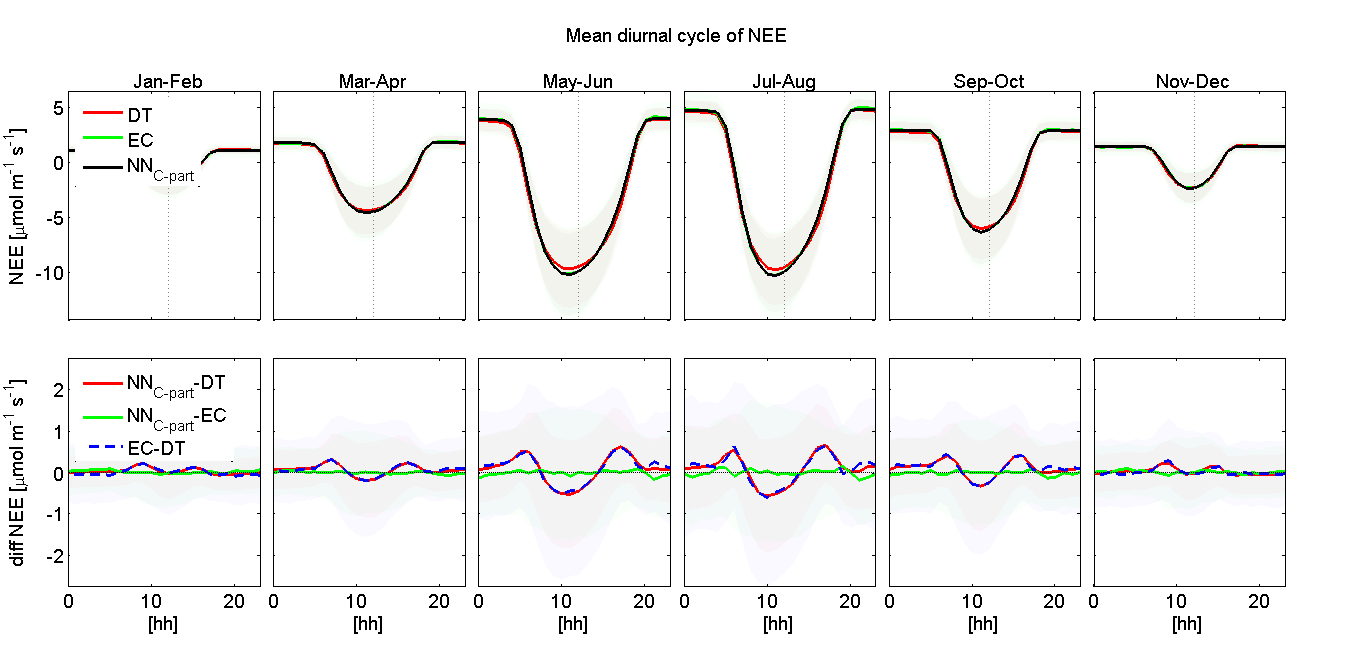


Figure S10: the top panels show the mean diurnal cycles of NEE fitted by NN_C-part_ (black line) and DT (red line) compared to the one of NEE measured by EC tower (green line). The bottom panels show the differences between diurnal cycles of NEEs: NN_C-part_ - DT (red line), NN_C-part_ - EC measurements (green line) and EC measurements - DT (dashed blue line). These patterns are related to study sites located in the Northern Hemisphere (latitude > 15°N) and at least 2-years of data.

**S6 Distribution of half hourly residuals among partitioned fluxes**


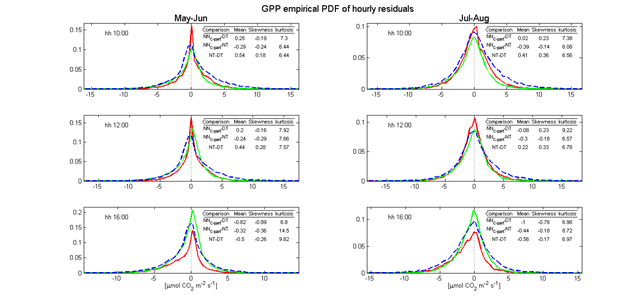


Figure S11: empirical probability density function of half hourly GPP residuals among partitioned methods in two different periods of the growing season and around three different hours: 10:00 a.m., 12:00 a.m. and 04:00 p.m. In red the residuals between NN_C-part_ and DT, in green the residuals between NN_C-part_ and NT and in blue the residuals between NT and DT. These patterns are derived from study sites located in the Northern hemisphere (latitude > 15°N).


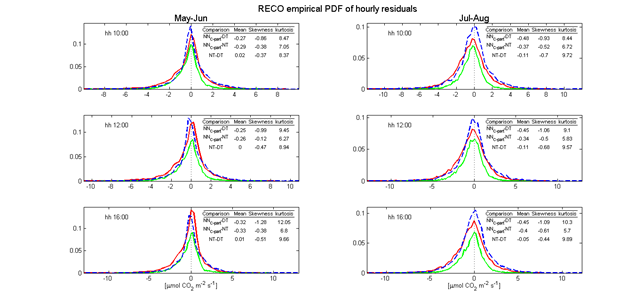


Figure S12: Same as figure s11 but for RECO.


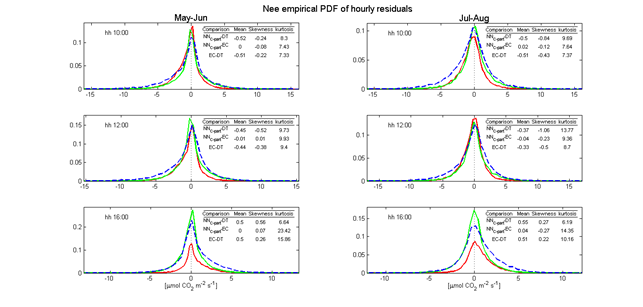


Figure S13: Same as figure s11 but for NEE.

**S7 Functional relationships between TS and SWC and RECO.**


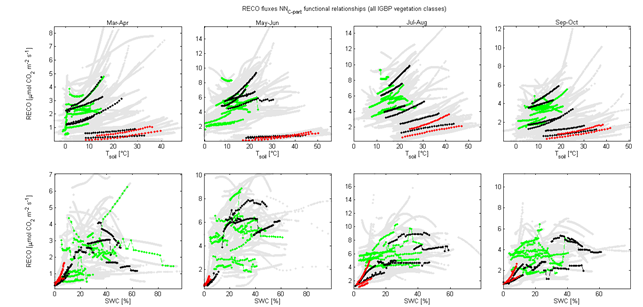


Figure S14: NN_C-part_ predicted responses of RECO as a function of the following micrometeorological drivers: a) soil temperature, ([°C], first row) and of b) soil water content, ([SWC], second row), simulated in the study sites of Northern hemisphere (latitude > 15°N) and in fixed seasonal conditions.

**S8 Importance of the drivers for RECO and GPP estimation in the NN_C-part_ approach**

In order to evaluate the importance of the single drivers used in the NN_C-part_ approach we permuted the order of observations one input at time (leaving the order invariant for the other features) and then we run the model in forward mode. The obtained output was then compared with the one produced without permutation of observations and the mean square deviation was retained as score of the importance (higher importance of the driver results in higher deviation to the standard run). This process was repeated 10 times for each driver and site-year; the results were summarized at site level by averaging the scores for each site year and then taking the median values across the 10 repetitions.

Although there is heterogeneity across the sites, the most important variables to predict the GPP resulted in the majority of cases: SW_IN, GPPprox and SW_IN_POT. VPD and air temperature also result among the important drivers in a number of sites while WD and SWC resulted in generally less important drivers except in a few sites (for example SWC is an important driver in dry and Mediterranean sites such US-Srg and IT-Ro1). It is also interesting to note that in sites where VPD is not ranked among the most important drivers, SWC or TA scale up their importance (different limiting factors). As reported, WD has generally low importance for almost all sites with the exception of a few study sites (e. g. DE-Obe, IT-Cpz) where this could be due to possible footprint heterogeneity.


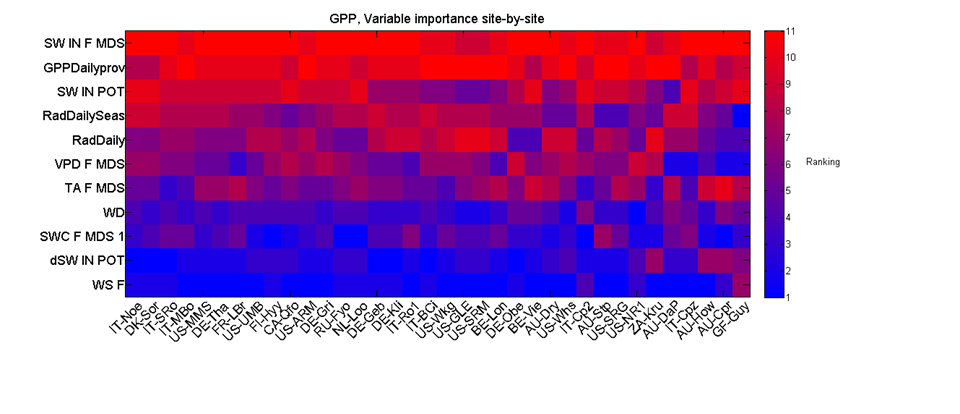


Figure S15: matrix of the ranking of drivers importance in the case of GPP. Drivers (rows) are ordered in relation to their variables importance expressed by their ranking (higher ranking means higher importance); In the columns there are the study sites ordered on the base of a clustering discriminatory analysis.

In the case of Reco, the most important drivers are represented by the average nighttime NEE, followed by the day of the year(DOY) meaning that the seasonality plays an important role. Among the variables that affect the diurnal variation of RECO, TS resulted generally more important than TA. SWC showed as expected higher importance in the Mediterranean study sites (IT-Ro1, IT-SRo) or in the dry ecosystems (e.g. US-Wkg, US-SRG).


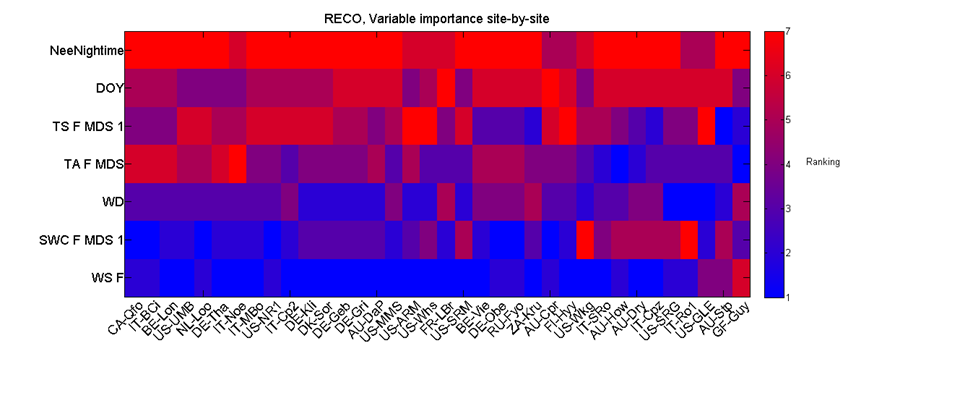


Figure S16: matrix of the ranking of drivers importance in the case of RECO. Drivers (rows) are ordered in relation to their variables importance expressed by their ranking (higher ranking means higher importance); In column there are the study sites ordered on the base of a clustering discriminatory analysis.

In order to evaluate the role of WD we analyzed the example of the IT-Cpz site that is characterized by a wind pattern toward East during daytime and toward West during nighttime and a vegetation structure that is not totally homogeneous. The difference between gross CO_2_ fluxes derived by NN_C-part_ using and not using in input wind variables is reported in figure S17 where in particular we see: a) the scatterplot (left side) among the half-hourly GPP (upper panels) and RECO (lower panels) estimates; b) the distribution of half hourly residuals (in the middle); c) the distribution of GPP and RECO residuals conditioned to the wind direction. Looking at the distribution of gross CO_2_ fluxes residuals, it is clear the effect of wind direction with negative values residuals mainly clustered in correspondence with the west direction (250-270 deg).


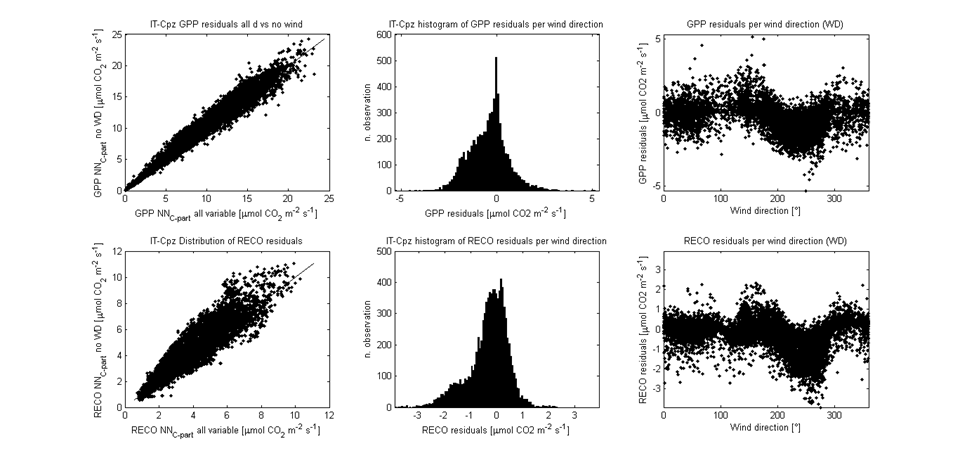


Figure S17: impact of wind on the partitioned fluxes in IT-Cpz study site. The residuals are derived by comparing the output from an ANN trained by including wind variables (WD and WS) among the input with another network that did not use these variables.

We extended the previous analysis (detailed reported for IT-Cpz) to all study sites used in this experiment (see Table 1 of the main text). For each site-year the systematic differences (MD) and the root mean squared differences (RMSD) among the NN_C-part_ settings (including vs excluding wind variables) were calculated. Table S2 shows a summary of results. Averaging results across study sites we found negligible effects of wind related variables on MD (the average value of MD was close to 0 and comparatively lower than average RMSD). However we found an appreciable spread across study sites for both the indices; also the spread of MD was comparatively higher than RMSD. This confirms the importance of wind related variables in specific sites.

Table S3: statistics that summarize the impact of the wind related variables to the estimates of the gross CO_2_ fluxes by NN_C-part_. Statistics were derived from the gross CO_2_ fluxes provided by NN_C-part_ by including and excluding wind related variables among the drivers. The average value of across site-year values and its standard deviation (in brackets) are reported. For the present analysis we used the gross CO_2_ fluxes estimates for the site year reported in the Table 1 of the main text.

| Flux | MD [µmol CO_2_ m^-2^ s^-1^] | RMSD [µmol CO_2_ m^-2^ s^-1^] |
| --- | --- | --- |
| GPP | -0,016 (0,143) | 0,838 (0,391) |
| RECO | -0,01 (0,15) | 0,63 (0,28) |

**Reference**

Ogée, J., Brunet, Y., Loustau, D., Berbigier, P. and Delzon, S. (2003), MuSICA, a CO2, water and energy multilayer, multileaf pine forest model: evaluation from hourly to yearly time scales and sensitivity analysis. Global Change Biology, 9: 697-717. doi:10.1046/j.1365-2486.2003.00628.x
